# U–Pb dating of volcaniclastic deposits from the Sinj Basin: implications for provenance and the tectono-sedimentary evolution of the External Dinarides

**DOI:** 10.1007/s00531-026-02567-w

**Published:** 2026-04-11

**Authors:** Robert Šamarija, Nevena Andrić-Tomašević, Oleg Mandic, Armin Zeh, Katja Mužek, Davor Pavelić, Matthias Schwotzer

**Affiliations:** 1 Institute of Applied Geosciences, Karlsruhe Institute of Technology, Adenauerring 20a, 76131 Karlsruhe, Germany; 2 Geological-Paleontological Department, Natural History Museum Vienna, Burgring 7, 1010 Vienna, Austria; 3 Department of Geology, Croatian Geological Survey, Sachsova 2, 10000 Zagreb, Croatia; 4 Faculty of Mining, Geology and Petroleum Engineering, University of Zagreb, Pierottijeva 6, 10000 Zagreb, Croatia; 5 Institute of Functional Interfaces, Karlsruhe Institute of Technology, Hermann-Von-Helmholtz-Platz 1, 76344 Eggenstein-Leopoldshafen, Germany

**Keywords:** Miocene, Zircon, Tephra deposition, Sediment reworking

## Abstract

In the Dinarides, a series of long-lived lakes, known as the Dinarides Lake System (DLS) developed during the Miocene. The Sinj Basin, situated in southern Croatia, is one of the best studied among them, providing valuable insights into the paleogeographic evolution of the region. Previous research established a chronostratigraphic framework in the NW part of the basin using paleomagnetic data calibrated by ^40^Ar/^39^Ar dating. Subsequent studies demonstrated that lacustrine flooding was diachronous. However, the influence of the Miocene Climatic Optimum (MCO) or the regional Miocene extensional tectonics is still debated. This study provides new constraints on the evolution of the Sinj Basin by integrating previous dating results with new LA–ICP–MS U–Pb–zircon dating of volcaniclastic and residual deposits from the NW, central, and SE parts of the basin. The obtained U-Pb ages range between ~ 17.7 and ~ 15.3 Ma and provide new constraints on the timing of initial lacustrine flooding. Correlation with the MCO suggests a close relationship between climate change and lacustrine development. Lake-level falls reflected by conglomerates and coals deposited in marginal and more distal parts of the basin, respectively, suggest environmental instability after ~ 15.3 Ma, towards the end of the MCO. Detrital zircon grains reveal a wide spectrum of ages from the Neoproterozoic to the Early Miocene, suggesting multiple redeposition cycles, starting with the Cadomian and Variscan orogenies, followed by Cretaceous–Oligocene uplift of the Dinarides, and the recycling of foreland basin deposits into the Miocene Sinj Basin. Finally, this study demonstrates the usefulness of LA–ICP–MS U–Pb dating for the reconstruction and correlation of isolated intramontane basins.

**Graphic Abstract:**

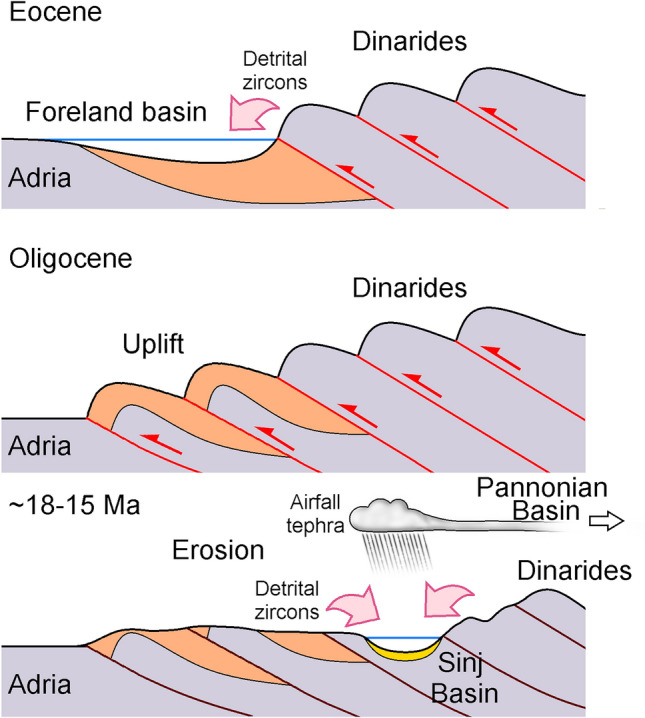

**Supplementary Information:**

The online version contains supplementary material available at 10.1007/s00531-026-02567-w.

## Introduction

**Fig. 1:**
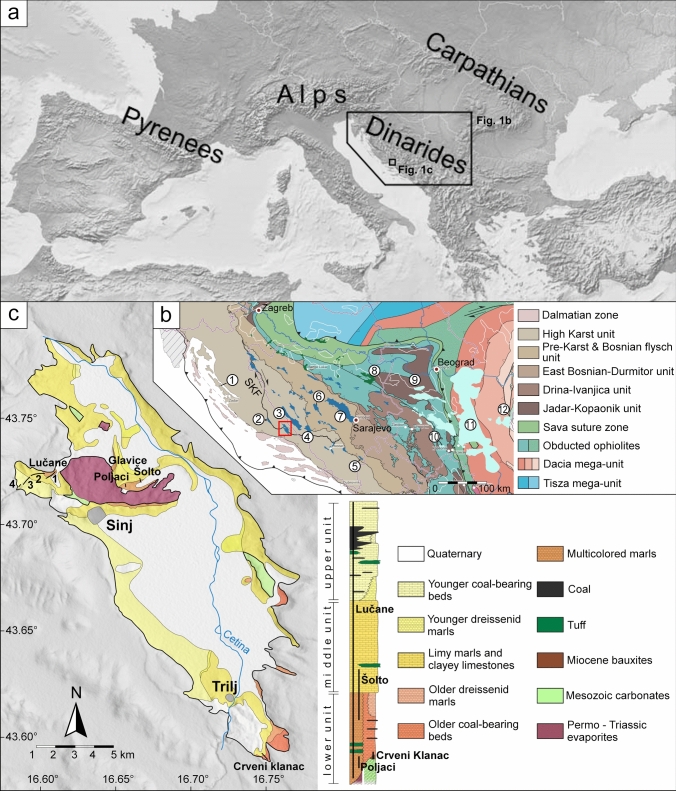
Maps showing the geographical position of the study area. **a** Position of the Dinarides and the Sinj Basin within Europe; **b** schematic geological map of the Dinarides modified after Schmit et al. (2020), showing the locations of Miocene paleolakes of the Dinarides (dark blue), Serbian (light blue) and Pannonian (green) basin systems. Individual basins mentioned throughout the text are labeled as follows: *1* Pag, *2* Drniš, *3* Livno, *4* Tomislavgrad, *5* Gacko, *6* Bugojno, *7* Sarajevo–Zenica, *8* Lopare, *9* Kolubara, *10* Ibar, *11* Morava, *12* Timok; SKF–Split–Karlovac fault; location of the Sinj Basin marked by red rectangle; **c** schematic geological map and lithostratigraphic column of the Sinj Basin, after Brlek et al. (2021), de Leeuw et al. (2010), Šušnjara and Sakač (1988), showing locations of investigated sections

Intramontane basins are integral parts of mountain ranges and record their depositional, erosional and deformational histories, as well as the evolution of their biota. Therefore, the study of intramontane basins offers a valuable source of information for paleoenvironmental, paleobiogeographic and tectonic reconstructions of mountain ranges. In the Dinarides, where nappe propagation and associated foreland basin deposition mostly ceased in the Oligocene (Korbar 2009; Mrinjek et al. 2012; Balling et al. 2021), intramontane basins represent the main source of information about the paleogeographic conditions during the Miocene. Moreover, these basins constrain the timing of different tectonic episodes in the Dinarides, as their deposits seal older structures while preserving younger, post-Middle Miocene deformations. Finally, the Miocene intramontane basins of the Dinarides preserve a detailed record of the Miocene paleoclimatic conditions in southeastern Europe. Recent research on the intramontane basins of the Dinarides suggested that their topography modified regional climatic conditions during the Miocene Climatic Optimum (MCO), by blocking the moisture from the Adriatic Sea and inducing dry conditions in the interior of the mountain range (Andrić-Tomašević et al. 2021). A subsequent compressional event (Andrić et al. 2017; Sant et al. 2018; Unen et al. 2019a, b), suggested to have occurred during the Late Miocene (~ 9 Ma, van Unen et al. 2019b), led to further modification of the landscape and climatic conditions by shifting the main topographic divide (highest topography peaks and ridges) towards the Adriatic Sea (Andrić-Tomašević et al. 2021). However, these hypotheses are still to be tested and geochronologic dating is vital for these tasks, as the resulting stratigraphic correlations provide a base for determining the timing of major tectonic events and paleoclimatic changes. An optimal place to study the interaction between tectonic and climatic conditions during mountain building is the Miocene Sinj Basin in southern Croatia (Fig. 1). A chronostratigraphic framework was first established for the NW part of the Sinj Basin (Lučane section) based on paleomagnetic data calibrated by ^40^Ar/^39^Ar dating of intercalated volcaniclastic deposits (de Leeuw et al. 2010). Subsequent zircon U–Pb dating by means of CA–ID–TIMS provided a better fit with the magnetostratigraphy (Brlek et al. 2021), demonstrating the advantages of U–Pb–zircon dating in diagenetically altered volcanic deposits. In addition, CA–ID–TIMS ages from bauxites in the SE part of the Sinj Basin (Crveni Klanac area), suggested that lacustrine flooding was diachronous (Brlek et al. 2021). However, due to restrictions of CA–ID–TIMS only a relatively small number of zircon grains could be dated. A small sample size may hinder the clear identification of the youngest volcanic zircon population, especially if a substantial inherited component is present. Therefore, the role of possible Miocene inherited zircon grains on the interpretation of the CA–ID–TIMS dating results is not entirely clear. Thus, to place new constraints on the evolution of the Sinj Basin, we present a comprehensive set of LA–ICP–MS U–Pb ages from a large number of well-characterized zircon grains from 11 samples, selected from the NW (Lučane section), central (Šolto section), and SE parts (Crveni Klanac area) of the Sinj Basin (Fig. 1). This set of data will: (1) provide new insights into the onset and progression of lacustrine flooding, (2) improve correlations of Sinj Basin lacustrine succession with Miocene climate changes and regional tectonic events, (3) place new constraints on the source of detrital zircons in the External Dinarides, and (4) help evaluate the influence of inherited Miocene zircon grains on the interpretation of the volcano-stratigraphic record.

## Geological settings

### Regional setting

The Dinarides are commonly subdivided into two parts: the External and the Internal Dinarides. The Internal Dinarides comprise composite nappes originating from the NE passive continental margin of the Adria microcontinent as well as obducted Neotethyan ophiolites (Schmid et al. 2008, 2020; van Hinsbergen et al. 2020), whereas the External Dinarides mostly consist of shallow-marine carbonates originating from the Mesozoic Adriatic carbonate platform (AdCP; Vlahović et al. 2005). The Dinarides formed as a result of the long-lasting convergence between Adria and Eurasia, beginning with Late Jurassic–Early Cretaceous ophiolite obduction (e.g., Mikes et al. 2008; Stojadinović et al. 2022b). This was followed by several successive stages of foreland basin development, which attest to the deformation of the NE Adria passive margin throughout the Cretaceous (e.g., Vranduk and Ugar fms.; Mikes et al. 2008; Schmid et al. 2008, 2020; Lužar-Oberiter et al. 2023; Bjelogrlić et al. 2026). The uppermost Cretaceous–Paleocene continental collision along the Sava suture zone resulted in propagation of the deformation front towards more external areas (Ustaszewski et al. 2010; van Unen et al. 2019a; Stojadinović et al. 2022a). The main phase of shortening and foreland basin development in the External Dinarides occurred during the Eocene–Oligocene, resulting in deposition of the Dalmatian “flysch” and the Promina “molasse” (Korbar 2009; Mrinjek et al. 2012). In the late Oligocene extension began in the Sava zone, driven by rollback (Schefer et al. 2011) or detachment of the Dinaric slab (Andrić et al. 2018). The subsequent Early–Middle Miocene extension affected a much wider area and was likely driven by the combined effects of the Hellenic and Carpathian slab rollbacks (de Leeuw et al. 2012; Andrić et al. 2017; Handy et al. 2019; Stojadinović et al. 2022a). During this time, the Dinarides (DLS) and Serbian (SLS) lake systems formed, flanking the western and eastern parts of the mountain range, respectively. The post-Middle Miocene development of the Dinarides was characterized by renewed compression and orogen-parallel transpressional wrenching induced by the continued northward motion and indentation of the Adriatic plate (Tari 2002; Andrić et al. 2017; Palenik et al. 2019; van Unen et al. 2019a,b). However, the magnitude of Miocene extension and subsequent compression and their relationships to the deposits of the DLS are still under debate and need to be tested.

### Sinj Basin

The Sinj Basin is situated along the Split–Karlovac fault (Chorowicz 1975), which represents a major zone of dextral transpression (Balling et al. 2021). It crosscuts the High-Karst thrust sheet of the External Dinarides and is interpreted to have accommodated along-strike differences in the amount and style of shortening in the (Balling et al. 2021). In the area of the Sinj Basin, it is characterized as an SSW-vergent thrust fault (Balling et al. 2021), exemplified by, e.g., the Svilaja Mts. to the west of Sinj, which occupy a hanging wall position (Papeš et al. 1982). The basin rims are composed of Mesozoic carbonates, middle–late Eocene “flysch,” late middle Eocene–Oligocene Promina “molasse,” and Oligocene Mosor breccias (Marinčić et al. 1969; Marinčić 1970; Mrinjek et al. 2012; Papeš et al. 1982), while in the central part, the basement of the lacustrine deposits is composed of Permo–Triassic evaporites (Šušnjara and Sakač, 1988; Šušnjara et al. 1992). The ~ 500 m thick Miocene lacustrine infill is dominated by freshwater carbonates (Vranjković et al. 2024). Based on paleontological and sedimentological data and geological mapping (Kerner 1905; Mandic et al. 2009; Marinčić et al. 1969; Neubauer et al. 2011; Olujić, 1999; Papeš et al. 1982; Šušnjara and Sakač, 1988; Vranjković, 2011; Vranjković et al. 2024) it is subdivided into a lower unit dominated by marls with coal intercalations and terrigenous input, a middle unit composed of mostly unfossiliferous limy marls and marly limestones, and an upper unit of clayey carbonates with coal intercalations. The succession is intercalated by several volcaniclastic layers (Šušnjara and Sakač, 1988; de Leeuw et al. 2010; Šegvić et al. 2014). Palynomorph data show that colder and humid climatic phases led to carbonate deposition, while warmer and more arid phases were related to the formation of coals (Jiménez-Moreno et al. 2008). A 2 m thick coal seam found in the upper part of the succession was formerly mined, and a large mammal fauna was collected from this layer by Olujić (1999). Breccia intercalations cropping out near the basin edge indicate a steep paleorelief (Mandic et al. 2009). There are three hypotheses suggested to explain the origin and evolution of the Sinj Basin: 1. strike–slip movements along the Split–Karlovac fault resulting in the formation of a pull-apart basin (Tari 2002; Mandic et al. 2009), 2. orogen perpendicular extension (de Leeuw et al. 2012; van Unen et al. 2019a, 2019b), and 3. dissolution of Permo–Triassic evaporites leading to collapse of overlying strata and the formation of a depression supplied by runoff and groundwater (Vranjković et al. 2024). Following deposition, the Sinj Basin experienced moderate compressional tectonics and minor counterclockwise rotation, as indicated by faults and folds, as well as by paleomagnetic data (de Leeuw et al. 2012; Jiménez-Moreno et al. 2008; Mandic et al. 2009; Marton et al. 2016; Olujić, 1999). Fossil bearing deposits of the Drniš Basin, located 60 km to the NW, correlate with the Sinj Basin and possibly belonged to one and the same large lacustrine depositional system (Neubauer et al. 2016). Both basins are presently separated by the Svilaja Mts. due to post-depositional thrusting (van Unen et al. 2019a, b).

Paleogeographically, Lake Sinj was a relatively shallow, carbonate hard-water lake with a flat bottom (Mandic et al. 2009; Vranjković et al. 2024). It formed part of the Dinarides Lake System, which occupied intramontane depressions in the western part of the Dinarides during the Early and Middle Miocene (Krstić et al. 2003; Harzhauser and Mandic 2008; de Leeuw et al. 2011; Mandic et al. 2020). Most of these basins accumulated thick infills, reaching ~ 900 m in the Bugojno Basin (Mandic et al. 2020), ~ 2.6 km in the Livno–Tomislavgrad Basin (de Leeuw et al. 2011), and over 3 km in the Sarajevo–Zenica Basin (Sant et al. 2018). This is interpreted to reflect relatively high subsidence rates related to extensional tectonics (Andrić et al. 2017; van Unen et al. 2019a, b). Lake formation and deposition also coincided with the MCO, representing a period of warm and humid climate conditions. Thus, climate dynamics might have played an additional, perhaps important role in their formation (Andrić-Tomašević et al. 2021; Brlek et al. 2021; de Leeuw et al. 2011, 2012).

### Previous dating results

The succession of the Sinj Basin was previously dated through integrated magnetostratigraphy and ^40^Ar/^39^Ar dating of three volcaniclastic layers from the Lučane section (de Leeuw et al. 2010), enabling correlation to the Astronomically Tuned Neogene Timescale (ATNTS; Lourens et al. 2004). These results suggested that deposition lasted between ~ 17.9 and ~ 15.0 Ma. However, correlation of the lowermost volcaniclastic layer, dated at 17.92 ± 0.18 Ma to the magnetostratigraphic data was problematic, due to intermittent outcrops covering the lowermost part of the succession and/or the diagenetic alteration of biotite crystals. Brlek et al. (2021), presented a revised age of 17.312 ± 0.024 Ma from the layer, using CA–ID–TIMS U–Pb dating of single zircon grains, indicating that lacustrine deposition started later than previously assumed. Additional CA–ID–TIMS zircon dating of a volcaniclastic horizon from the Glavice section and of bauxites from the Crveni Klanac section suggested that lacustrine flooding was diachronous, reaching the SE basin margin by 17.0 Ma (Brlek et al. 2021, 2023). However, the lowermost ash layer from the Lučane section remained undated, leaving uncertainties in the age of the oldest lacustrine deposits. Furthermore, the ages presented by Brlek et al. (2021, 2023, 2024a; b) indicate that the zircon populations in volcaniclastic layers and bauxites of the Sinj Basin contain inherited components, suggesting mixing of zircon populations from different sources.

## Description of investigated sections

**Fig. 2:**
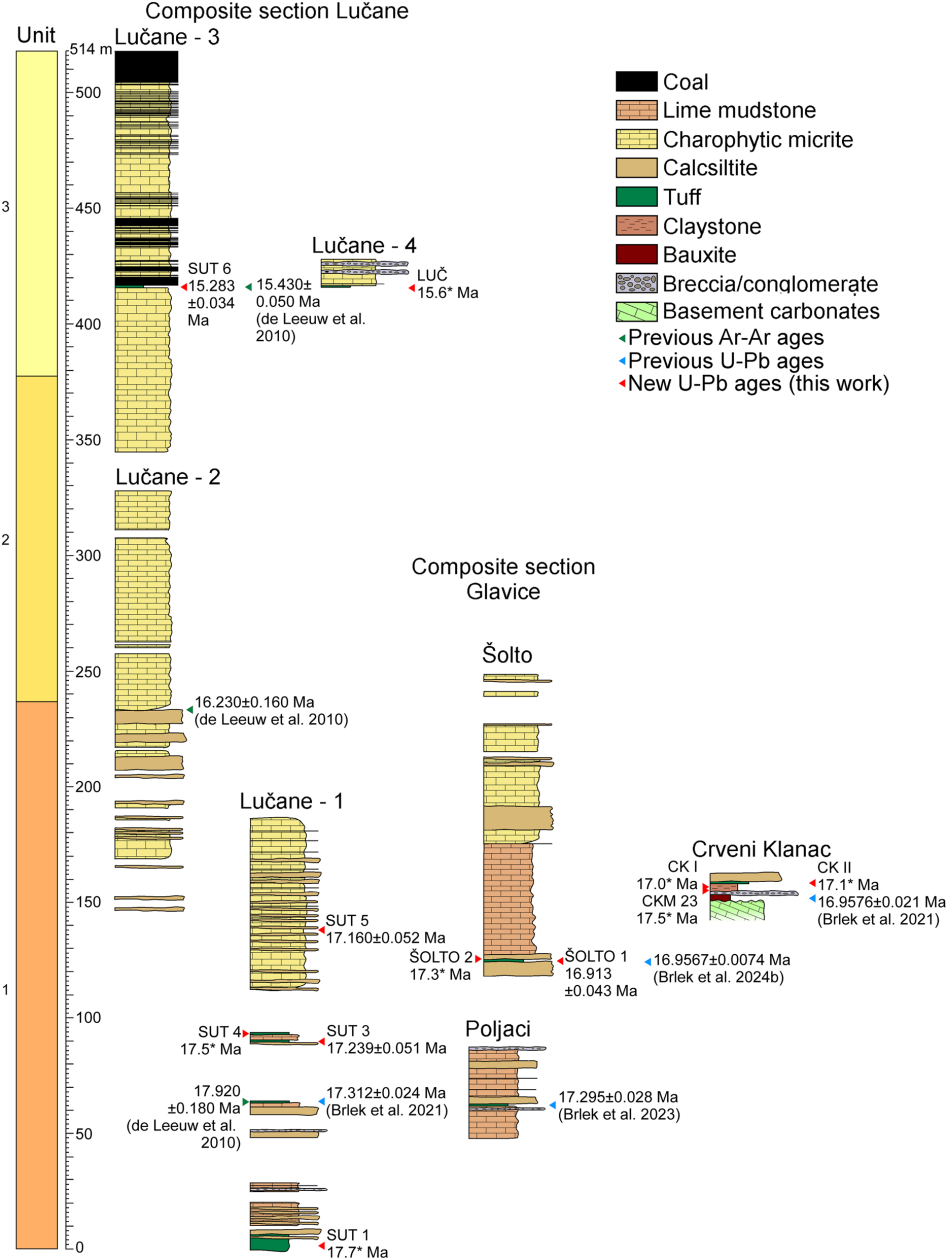
Stratigraphic profiles with volcaniclastic layers of the Sinj Basin summarizing (modified after Vranjković et al. 2024). Ages are from this and previous studies (U–Pb–zircon ages of Brlek et al. 2021, 2023, 2024a, b and ^40^Ar/^39^Ar ages of de Leeuw et al. 2010). Ages marked with * represent reworked volcaniclastic deposits (see discussion), and are based on the predominant Miocene age peaks of the respective samples

The 514-m-long Lučane composite section is located in the westernmost part of the Sinj Basin (Fig. 1), along the eastern limb of an NE–SW striking syncline (Olujić, 1999; de Leeuw et al. 2010). It has previously been described in detail by Jiménez-Moreno et al. (2008); Mandic et al. (2009); de Leeuw et al. (2010); and Vranjković et al. (2024). This section encompasses all three lithological units of the basin infill (Figs. 1, 2). The contact with the underlying Jurassic limestones is covered by Holocene alluvial deposits and can be observed to the NE of the section (Papeš et al. 1982; de Leeuw et al. 2010). The section begins with a prominent, so far undated volcaniclastic horizon, followed by calcareous mudstones interpreted to have been deposited in the distal part of the lake (Vranjković et al. 2024). Calcsiltite, coal, and volcaniclastic intercalations are relatively common (Fig. 1). The volcaniclastic layer dated at 17.312 ± 0.024 Ma by Brlek et al. (2021) occurs approximately 60 m above the base of the section. From approximately 100 m upwards, a prominent facies change occurs. Strata above this level consist of alternating light gray micrites and dark, bioclastic wackestones. Bioclasts mostly consist of encrusted charophyte stems and fruits of the aquatic plant *Damasonium sutinae* (Mandic et al. 2009; Vranjković et al. 2024). These limestones are interpreted to have formed in a low-energy littoral environment, corresponding to a carbonate bench platform situated near the lake margin (Vranjković et al. 2024). Calcsiltite intercalations are still common up to approximately 230 m, after which limestones become the dominant lithology. A volcaniclastic layer dated at 16.23 ± 0.16 Ma by de Leeuw et al. (2010) occurs above the uppermost calcsiltite intercalation. Another prominent volcaniclastic layer, dated at 15.43 ± 0.05 Ma by de Leeuw et al. (2010), is present at ~ 416 m, just beneath the start of the coal-bearing series. Upwards coal intercalations become increasingly common, suggesting the relative proximity of a vegetated shore (Vranjković et al. 2024). The “main,” 2 m thick coal layer from which mine workers collected large mammal remains occurs at approximately 512 m in the section (Olujić, 1999; Mandic et al. 2009; de Leeuw et al. 2010).

Approximately 250 m SW of the Lučane 3 subsection (Fig. 2), at the western limb of the syncline, a ca. 10 m thick section is exposed, termed the Lučane 4 subsection. It consists of limestones with prominent breccia and conglomerate lenses, interpreted to have formed in close proximity to the lake margin and comprising clasts from older marginal lake deposits (Mandic et al. 2009; Olujić, 1999; Vranjković et al. 2024). These clastics are underlain by a prominent volcaniclastic layer not dated so far. The composite Glavice section is located in the central part of the basin. It comprises two subsections, which cover parts of the lower and middle lithological units of the Sinj Basin infill (Figs. 1, 2). The basement is composed of Permo–Triassic evaporites, cropping out to the south (Šušnjara et al. 1992). The lower, Poljaci subsection is composed of calcareous mudstones with calcsiltite intercalations. A volcaniclastic layer from the Poljaci subsection yielded a CA–ID–TIMS zircon age of 17.295 ± 0.028 Ma (Brlek et al. 2023). At the top of the Poljaci subsection, a prominent carbonate breccia occurs, made up mostly of angular clasts and large boulders comprising Jurassic, Cretaceous to Paleogene limestones and dolomites (Vranjković et al. 2024). The upper, Šolto subsection is located about 1 km to the east of the Poljaci subsection. The thickness of the covered interval separating these two subsections is estimated at 30 m (Vranjković, 2011). The base of the Šolto section is composed of calcsiltites intercalated by two volcaniclastic layers. It continues with calcareous micrites grading upwards into micritic limestones of the bench platform (Vranjković et al. 2024). Several more prominent calcsiltite intervals occur in the upper half of the section. The Crveni Klanac section is located in the SE part of the basin, in the vicinity of the town Trilj (Fig. 1). Here, the basement is composed of Mesozoic carbonates, immediately overlain by a prominent bauxite deposit laterally bounded by steep-dipping faults (Brlek et al. 2021; Šušnjara and Sakač, 1988). Its formation has been related to the MCO and dated at 16.9576 ± 0.021 Ma by Brlek et al. (2021). The section continues with a laterally thinning breccia lens, followed by reddish to grayish bauxites with plant root remains. The very top of the section is composed of a whitish limestone marking the transition to perennial lake conditions.

## Analytical techniques

**Fig. 3:**
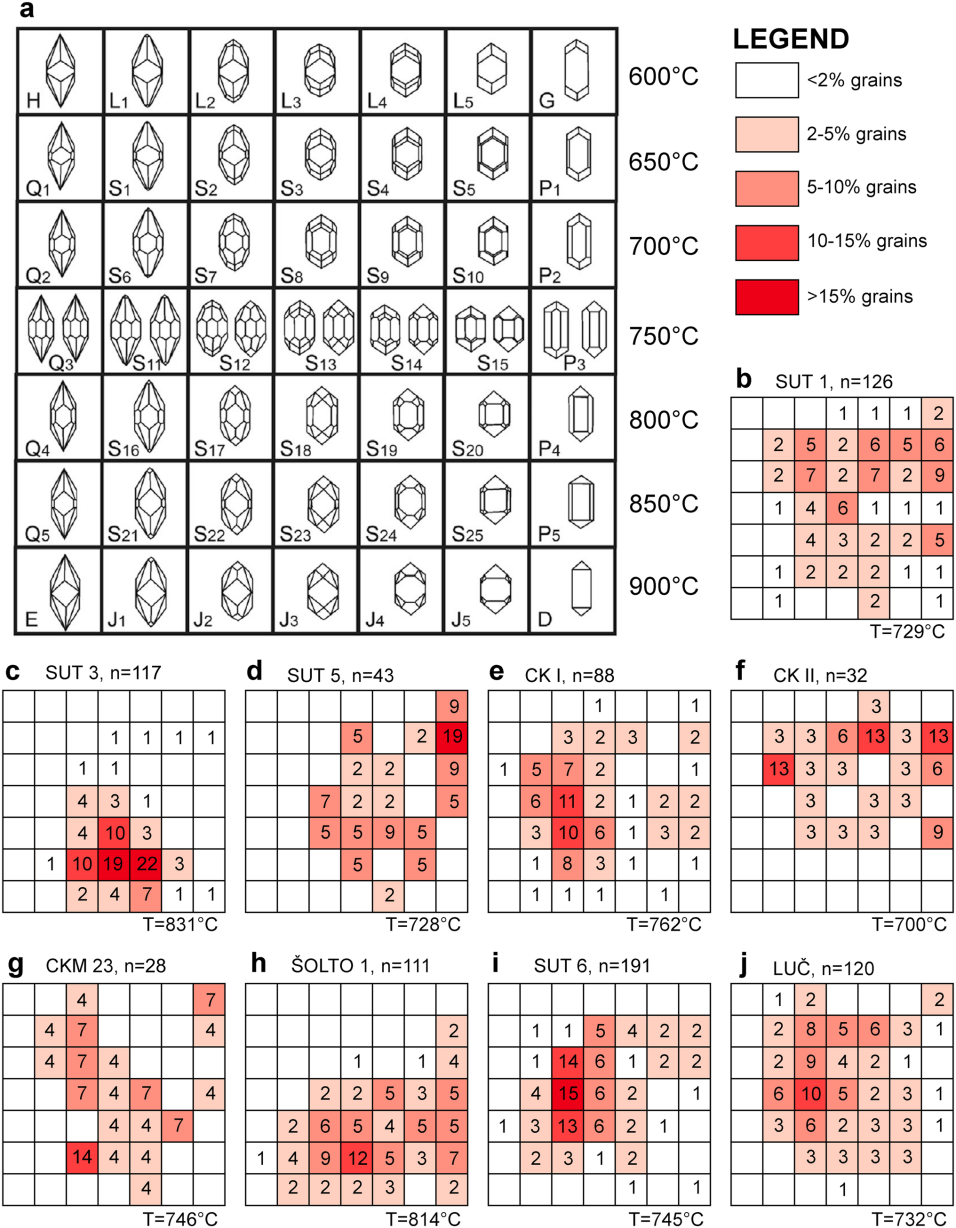
Typologies and average temperatures of zircon populations in volcaniclastic layers of the Sinj Basin. **a** Typology diagram according to Pupin (1980); **b–j** distribution of zircon typologies in the investigated volcaniclastic samples. Only samples with > 20 angular zircon grains are shown. The average temperature (T) is calculated using the methodology described in Pupin (1980)

**Fig. 4:**
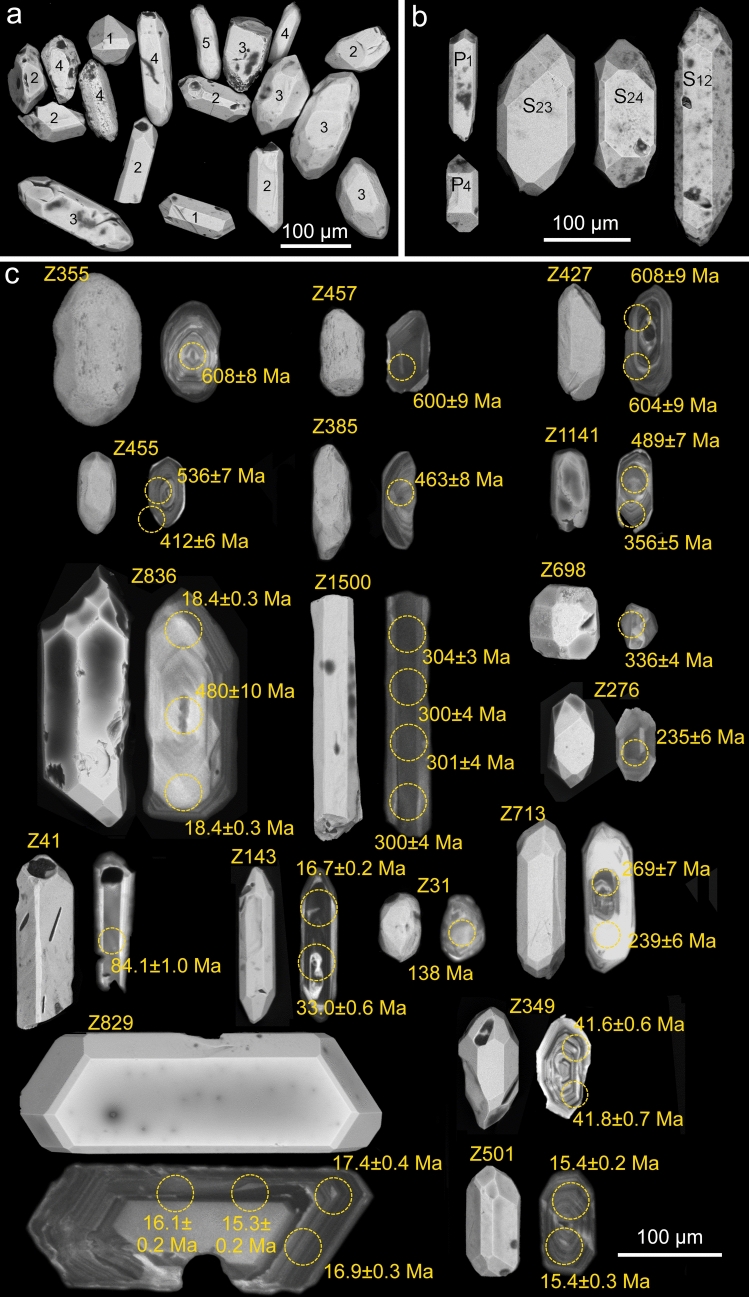
Common external morphologies and internal zoning patterns of the analyzed zircon grains. **a** SEM images of zircon grains (CK I sample) displaying all degrees of roundness (DOR) categories (Zeh et al. 2021; *1* perfect euhedral, *2* slightly rounded, *3* edges significantly rounded, *4* relic primary faces, *5* completely rounded). **b** SEM images of common zircon typologies (after Pupin 1980). **c** CL and SEM images showing internal zoning patterns of Miocene zircons and associated external morphologies. For details, see the discussion

Samples of volcaniclastic and residual deposits were collected from profiles in different parts of the Sinj Basin (Figs. 1, 2). Several kilograms per sample were collected for heavy mineral separation. All samples were dried at < 90 °C, crushed with a jaw crusher, and a disc mill for 120 s, and about 0.5–1.0 kg powder was washed in a gold-wash pan. Individual zircon grains were handpicked under ethanol, pipetted on double-sided adhesive tape, sputtered with Au for 15 s, and imaged for their morphologies by scanning electron microscopy (SEM) using a TESCAN VEGA2 electron microscope at the Department of Mineralogy and Petrology at the Karlsruhe Institute of Technology (KIT), Germany. The SEM images were used to determine zircon typologies, average zircon formation temperatures, and the degree of roundness-DOR (Pupin 1980; Zeh and Cabral 2021). Subsequently, the grains were embedded in epoxy, ground to expose their center parts, and investigated by cathodoluminescence (CL) imaging to gain information about the internal zoning patterns. The CL images were produced using a Thermofisher Scientific Quattro S Environmental Scanning Electron Microscope (ESEM) at the Institute of Functional Interfaces at KIT. Finally, all grains were numbered and the Degree of Roundness (DOR) and typologies determined, applying the classification scheme of Zeh and Cabral (2021) and Pupin (1980), respectively. The results are shown in Figs. 3, 4, and Table 1.

**Table 1:** Results of U–Pb dating and summary of dated and inherited zircon grains per sample

Sample	N (all)^a^	N (i)^b^	% inherited	PDD Age (Ma)^c^	N (calc)^d^	Age (Ma)^e^	MSWD, Prob.^f^
SUT 6	88	3	3%	15.3	89	15.283 ± 0.034	1.2, 0.14
LUČ	114	92	81%	15.6		15.6	
SUT 5	48	2	4%	17.1	44	17.160 ± 0.052	1.3, 0.1
SUT 4	61	49	80%	17.5		17.5	
SUT 3	50	2	4%	17.2	44	17.239 ± 0.051	1.3, 0.07
SUT 1	85	52	61%	17.7		17.7	
ŠOLTO 2	36	33	92%	17.3		17.3	
ŠOLTO 1	52	3	6%	17.0	47	16.913 ± 0.043	1.4, 0.03
CK I	76	41	54%	17.0		17.0	
CK II	56	29	52%	17.1		17.1	
CKM 23	31	18	58%	17.5		17.5	

^a^Total number of analyzed grains

^b^Number of inherited grains (i)

^c^Age of main Miocene probability density distribution peak

^d^Number of spot analyses used for weighted mean age calculation

^e^Weighted mean age

^f^MSWD, Prob.—Mean standard weighted deviation, Probability of fit

**Fig. 5:**
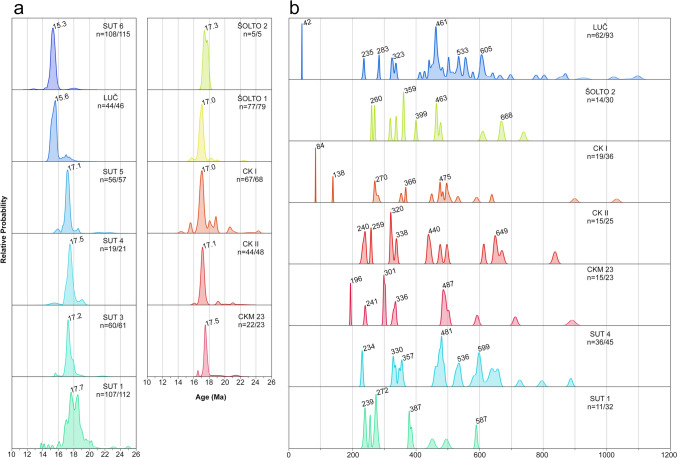
Age spectra of individual zircon ages from samples collected in the Sinj Basin. The data are presented in age vs. relative probability diagrams. **a** Miocene analyses from all samples. Numbers denote the ages of the predominant Miocene population. Analyses with a concordance level of 1–199% were used. **b** Inherited zircon spectra for group II samples (see discussion), showing the major age peaks. Data within a 90–110% concordance range were plotted

**Fig. 6:**
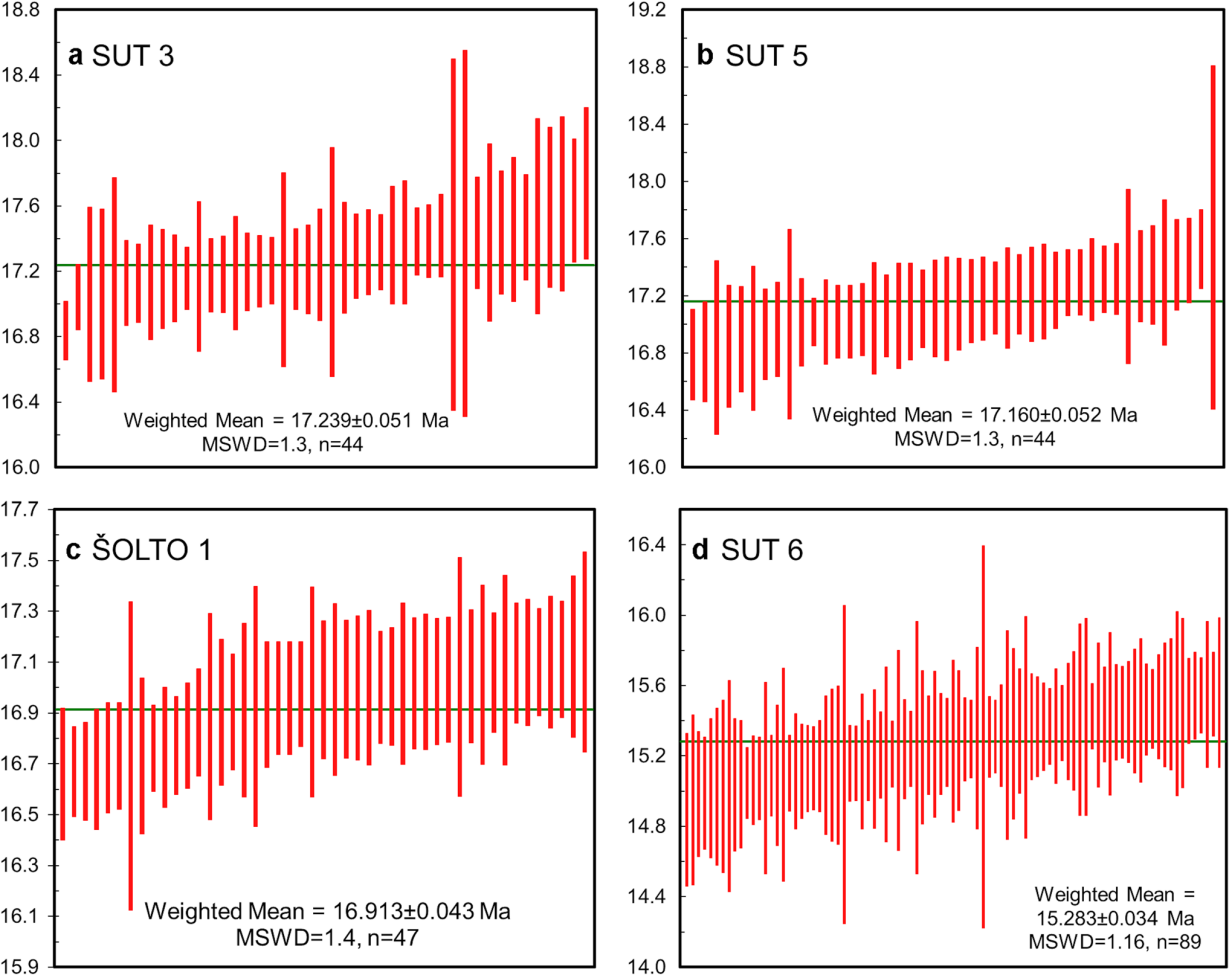
Results of weighted mean age calculations for the group I samples. Data-point error symbols are 2σ. **a** SUT 3 sample, Lučane-1 subsection; **b** SUT 5 sample, Lučane-1 subsection; **c** ŠOLTO 1 sample, Šolto subsection; **d** SUT 6 sample, Lučane-3 subsection

Uranium–Th–Pb analyses were performed by laser-ablation sector-field inductively coupled mass spectrometry (LA–SF–ICP–MS) during three sessions, using a 193 nm ArF Excimer laser (Analyte Excite +, Teledyne Photon Machines) coupled to a Thermo-Scientific Element XR instrument at KIT. Zircon grains of unknown age were analyzed together with the reference zircon BB (primary reference material), as well as Plešovice, KA, and SING (secondary reference material). From each sample between 31 and 114 zircon grains were selected for U–Th–Pb analyses (Table S2). CL images were used to avoid inclusions, fractures, and alteration zones, and to detect multiple crystallization domains (cores and rims). Spot analyses were mostly placed on the tips of zircon grains to obtain the youngest possible crystallization ages. On zircons with distinct core–rim relationships, several laser spot analyses were performed to identify possible age variations (Fig. 4). Detailed information about instrument conditions is presented in electronic supplement materials (Table S1), and the results of reference zircon measurements and unknowns can be found in Table S2. All raw data were corrected offline using an in-house MS Excel© spreadsheet program (Gerdes and Zeh 2006, 2009). A common Pb correction based on the interference and background corrected ^204^Pb signal and a model Pb composition (Stacey and Kramers 1975) was only applied to inherited grains older than 200 Ma when the common Pb uncorrected age was significantly outside the error of the corrected age. Age spectra of zircon populations are plotted with the freeware AgeDisplay (Sircombe 2004), using ^206^Pb/^238^U ages for analyses < 1000 Ma, and ^207^Pb/^206^Pb ages for analyses > 1000 Ma. Analyses with ages > 25 Ma plotted within a 90–110% concordance interval were selected to avoid possible mixing of multiple age domains or Pb loss, whereas younger analyses were plotted within a 1–199% concordance interval (Fig. 5). The ages of volcaniclastic zircon populations are presented in ranked isotope plots (Fig. 6) which for most samples comprise data produced during two or even three different analytical sessions (Table S2). Weighted mean ^206^Pb/^238^U ages were calculated for the Miocene zircon populations, only including analyses with concordance level between 1 to 199%. For all analyses, the concordance level is calculated from the (^206^Pb/^238^U age)/(^207^Pb/^206^Pb age) × 100 (ESM S2). After rejecting young outliers, assumed to result from Pb loss (see discussion), weighted mean ages were calculated by including successively older analyses until a low-MSWD threshold was reached (Wendt and Carl 1991). Weighted mean ages are presented for samples, where significant post-depositional reworking can be excluded based on petrological constraints and inherited zircon content (Table 1; Fig. 5; see discussion). All ages were calculated using the software ISOPLOT v.3.75 (Ludwig 2012). For reworked samples, the age of the predominant Miocene age peak is shown (Fig. 5a; Table 1).

## Results

### Zircon shape parameters and crystallization temperatures

Zircon populations were dated from eight volcaniclastic and three residual deposit samples from different profiles in the Sinj Basin (Fig. 2). An analysis of zircon shape parameters reveals significant differences among the samples (Figs. 3, 4; Table 1; Tables S2, S3). Most samples are dominated by angular magmatic zircon grains (average DOR = 1.8–2.2), except for five samples (CK I, LUČ, ŠOLTO 2, SUT 4 and CKM 23) which have an av. DOR = 2.5–3.7. These contain a larger number of rounded grains, pointing to a detrital origin (Fig. 4). Zircon grains of most samples show a wide range in typologies, and average formation temperatures (av.T = 700–814 °C), except sample SUT 3 revealing a limited cluster around typologies S23–S24 (av.T = 831 °C; Fig. 3c). Considerable overlap exists among the samples CK I, SUT 1, SUT 6 and LUČ, dominated by the typologies S7–S8, S12–S13, and S17–S18 (av. T = 729–762 °C), and among sample SUT 5 and CKII with a prominent cluster at typology P1 (av. T = 700–728 °C). The samples CKM 23 and ŠOLTO 1 are somewhere in between (Fig. 3, Table 1).

### U–Pb–zircon dating

**Fig. 7:**
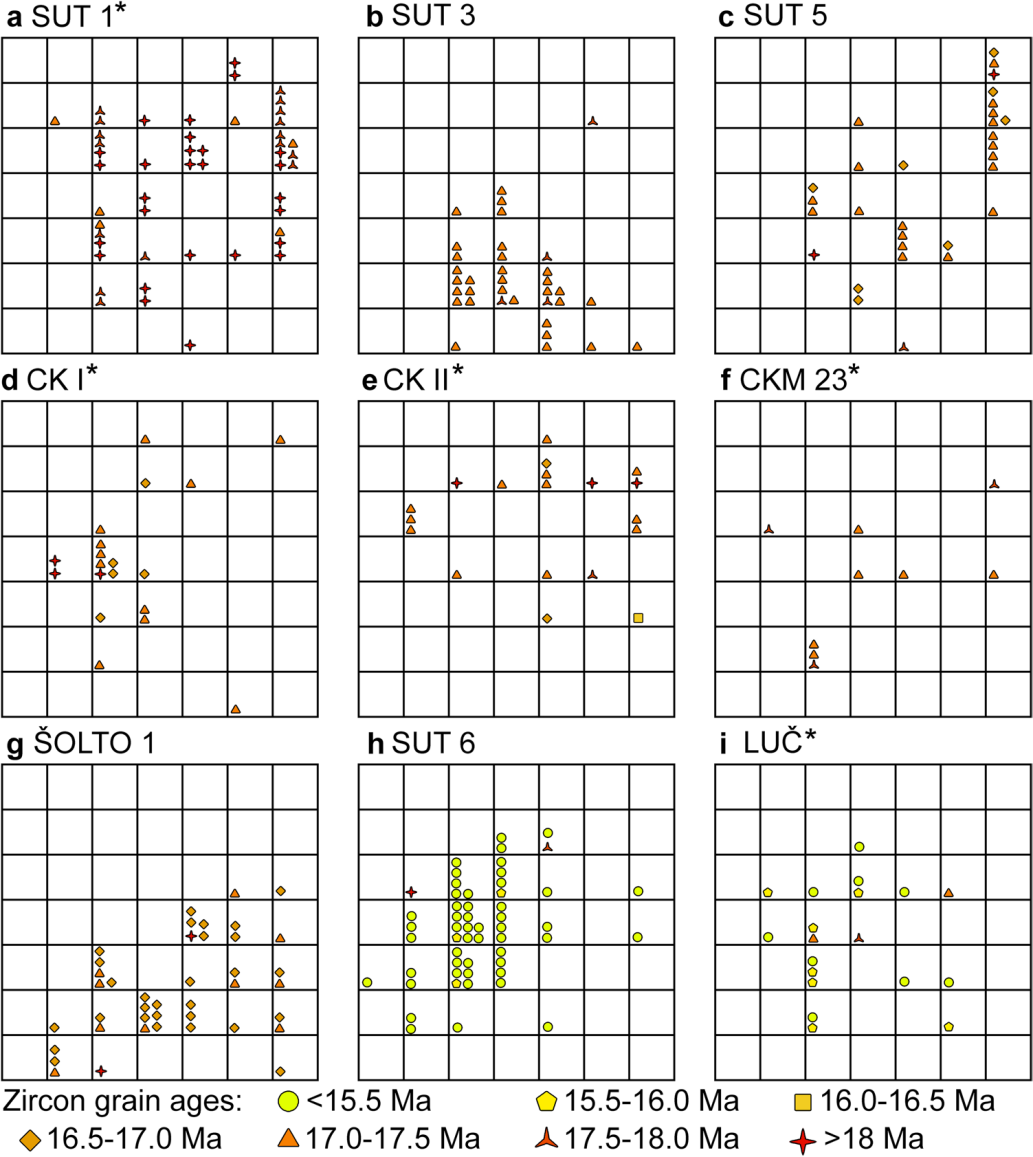
Typologies of dated Miocene zircons for selected samples (**a–i**), following the typological scheme as in Fig. 3. Zircons are subdivided into 0.5 Ma age increments, and plotted according to their typology. Asterisks mark group II samples (see discussion)

Zircon grains from most samples show a wide range of ages from the Miocene to the Archean (Figs. 5, 6; Table 1). Considering the maximum age of the Sinj Basin (de Leeuw et al. 2010), we consider the analyses with ages > 18 Ma to be inherited. In all samples there is a clear gap between the predominant Miocene age peak and the, respectively, older ones, which facilitates the separation of the Miocene from the inherited zircon populations in the probability density diagrams (Fig. 5). The samples may thus be subdivided into two groups based on inherited zircon content. Samples of group I contain ≤ 6% of inherited grains (SUT 3, SUT 5, SUT 6 and ŠOLTO 1), whereas samples of group II contain > 50% of inherited grains (SUT 1, SUT 4, LUČ, ŠOLTO 2, CKM 23, CK I, CK II). Inherited zircon grains were found in all samples, independently whether these were collected from the northwestern, central and southeastern part of the basin. Age spectra from group II samples contain rare Archean to Paleoproterozoic (2568–1964 Ma), Neoproterozoic (1000–530 Ma), Cambro–Ordovician (530–444 Ma), Devonian–Carboniferous (398–310 Ma) and Permian (295–251 Ma) and Triassic (cca. 240 Ma) zircon populations (Fig. 5b; Table S2). In addition, rare zircon grains of Jurassic–Cretaceous age (cca. 196, 138 and 84 Ma) were found at the Crveni Klanac section. An Eocene age (42 Ma) was only found in sample LUČ. All samples provided Miocene ages, comprising up to 97% of the analyzed grains (Table 1). The probability peaks for these Miocene ages range between 17.7 to 15.3 Ma (Fig. 5a). The oldest and youngest peaks were obtained from the stratigraphically lowest and, respectively, highest volcaniclastic layers in the Lučane section (Fig. 2). Finally, in a few samples, some zircon grains, yield ages significantly younger than the predominant Miocene population (e.g., sample CK I; Figs. 4, 5). In most samples the typologies of Miocene zircon grains form relatively tight clusters (Fig. 7). There is no clear link between Miocene zircon age and typology, except perhaps in sample SUT 1 (see discussion).

### Zircon textures

Most of the investigated zircons display oscillatory, sector and/or banded internal zoning patterns in CL images suggesting a magmatic origin (e.g., grains Z 349, Z355, Z427, Z457, Z501, and Z1500; Fig. 4c). Only a few grains display inherited cores, revealed by abrupt changes in brightness and/or zoning disruption (e.g., grains Z836, Z713, Z1141, and Z143; Fig. 4c). Such core–rim relationships point to multiple episodes of zircon growth in, respectively, younger magmatic systems, which is also reflected by significant age differences between the cores and rims (e.g., grain Z836: core = 480 Ma, rim = 18.0 Ma; grain Z143: core = 33 Ma, rim = 16.7 Ma; Fig. 4c). Some grains also show complex zoning patterns and variable, non-overlapping Miocene ages (e.g., grain Z829 = 15.3–17.4 Ma; Fig. 4c).

## Discussion

### Depositional mechanisms of volcaniclastic horizons

**Fig. 8:**
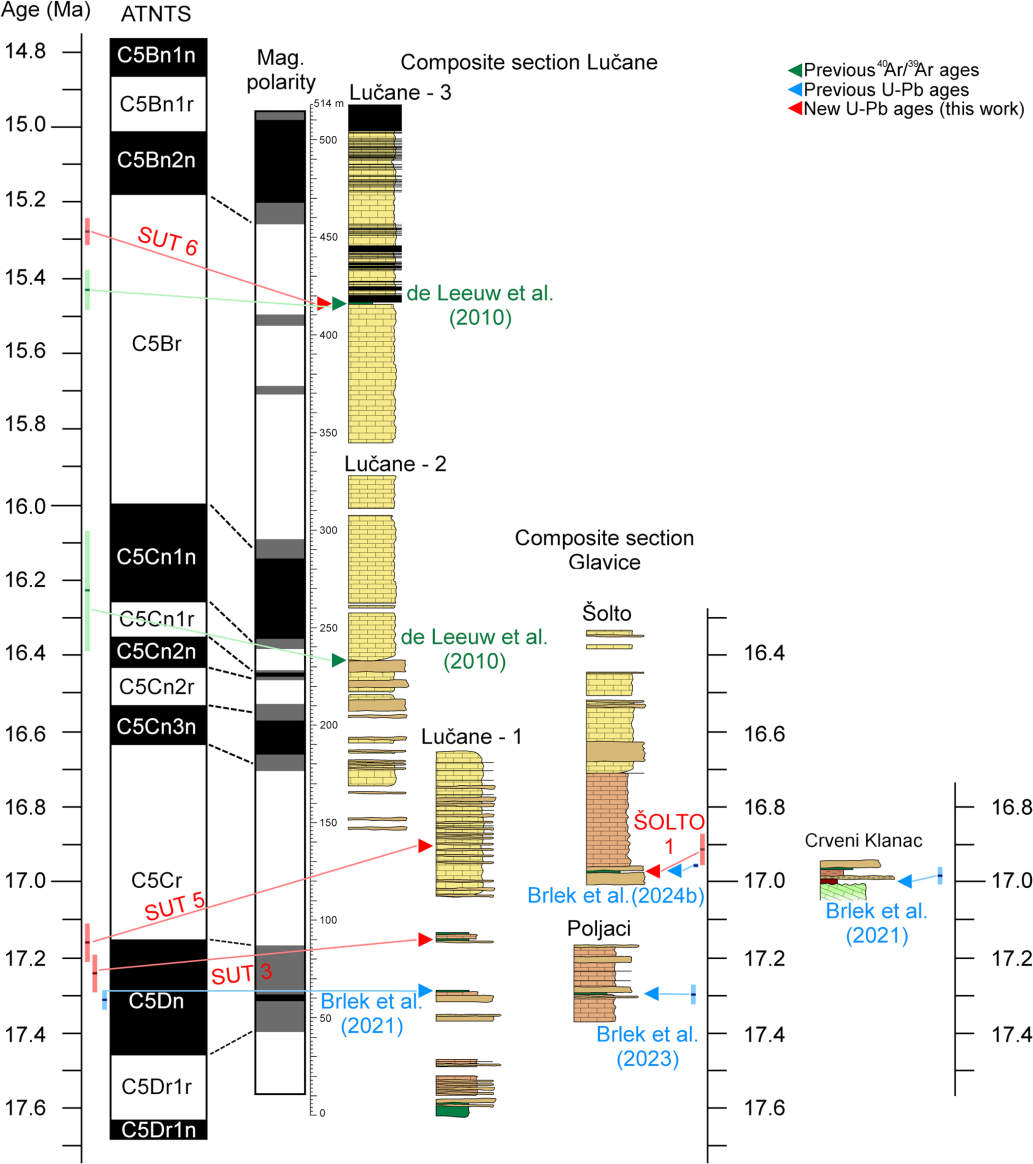
Age model for the Sinj Basin based on radiometric ages from this and previous studies (U–Pb–zircon data of Brlek et al. 2021, 2023; and ^40^Ar/^39^Ar data of de Leeuw et al. 2010), and magnetostratigraphy (de Leeuw et al. 2010), calibrated to the ATNTS (Raffi et al. 2020)

The results of this study indicate that the Miocene zircon populations in volcaniclastic and residual deposits from the Sinj Basin become systematically younger from the bottom to the top of the succession and suggest that deposition occurred over a period of 2.4 Myr (Figs. 2, 8). Inherited pre-Miocene zircon grains that occur in all samples indicate that the investigated volcaniclastic and residual deposits contain volcano–sedimentary detritus derived from a variety of sources. Thus, for interpretation of the ages it is important to know when the zircons from different sources were mixed with each other. In general two end-member scenarios might be considered: (1) all zircon grains, Miocene volcanic and inherited, stem from the same magma chamber and were transported by the same tephra cloud and (2) Miocene magmatic and inherited grains were mixed together during post-air-fall sediment transport (i.e., reworking). In the first scenario, the youngest volcanic zircon grains represent the time of deposition, and in the second scenario a maximum depositional age, more or less close to the time of deposition. In this context, it is pertinent to note that the investigated volcaniclastic deposits display stark differences in the amount of inherited zircon grains. Samples of group I contain ≤ 6% of inherited grains and are composed either of altered vitroclastic tuffs (SUT 6 and ŠOLTO 1) or tuffaceous clays (SUT 3 and SUT 5; Šegvić et al. 2014). In contrast, samples of group II contain > 50% of inherited grains and are made up either of tuffaceous clays (SUT 1 and SUT 4; Šegvić et al. 2014) or bauxites (CK I, CK II, CKM 23; Brlek et al. 2021). For the samples of group I, unambiguous evidence for post-depositional reworking is only reflected by sample SUT 5 containing characean oogonia (Šegvić et al. 2014). For the samples of group II, post-depositional reworking is commonly reflected by petrographic observations (e.g., silt–clay interlayering, non-volcanic minerals, and bauxitization) and by the large number of inherited zircon grains. The existence of tuffaceous clastites with abundant detrital zircon grains in the Sinj Basin was already noted by Šušnjara and Šćavničar (1974). The volcaniclastic horizons of the samples SUT 1 and SUT 4 were, moreover, observed to contain detrital chlorite and garnet grains (Šegvić et al. 2014). The precursor material, from which the samples CK I, CK II and CKM 23 were taken, likewise consists of a mix of siliciclastic detritus and volcaniclastic material of Oligocene–Miocene age (Šušnjara and Šćavničar, 1976, 1978; Brlek et al. 2021). Although no petrographic observations exist for the samples ŠOLTO 2 and LUČ, the large proportion of inherited zircon grains and the high degree of zircon roundness (see Tables S2e, S3) indicate post-depositional reworking. We note that sample LUČ has been sampled close to the margin of the basin, which was characterized by fluvial and/or debritic coarse clastic input (Mandic et al. 2009; Vranjković et al. 2024). It is most likely that the inherited zircons were supplied from sedimentary rocks exposed around the Sinj Basin, comprising Lower Triassic siliciclastics, Middle Triassic volcano-clastics, Eocene “flysch,” Eocene–Oligocene Promina deposits and Oligocene Mosor breccias (see section: provenance of detrital zircon grains). Therefore, the strata from which the group II samples were collected likely resulted from re-mobilization of air-fall tephra by rivers and/or floods (cf. Brlek et al. 2024a, b), which led to mixing of Miocene age air-fall tuffs with siliciclastic detritus before their deposition by gravity flows. Some of the older zircon grains in the investigated volcaniclastic layers might on the other hand represent xenocrysts, which were transported with the tephra clouds from the place of eruption (magma chamber) towards the Sinj Basin. Evidence for this option is provided by zoned zircon grains with inherited cores (Fig. 4), i.e., analyses which yield core ages which are within error significantly older than the rim age. Taking all information into account, it is most likely that the youngest volcanic zircon populations found in group I samples (SUT 3, SUT 5, SUT 6 and ŠOLTO 1) date the timing of ash layer deposition, whereas those in group II samples CK I, CK II, CKM 23, SUT 1, SUT 4, LUČ and ŠOLTO 2 reflect maximum depositional ages (MDAs), more or less close to the time of tephra air fall.

### Zircon typologies and ages

In addition to the available petrological constraints, it is important to note that the typologies of Miocene grains in group I samples form relatively tight clusters (Fig. 7b, c, g, h), suggesting that the investigated grains originated from one instance of crystal growth. Group II samples mostly show similar typological variations, and no clear link between Miocene zircon age and typology could be established (Fig. 7a, d, e, f, i). We further note that samples CK I, CK II, SUT 5 and ŠOLTO 1 display different predominant typologies (Figs. 3, 7), despite the similar ages (Fig. 5). This suggests that all four samples probably represent products of distinct volcanic episodes. Only sample SUT 1 shows a slight correlation between zircon age and typology. Most grains > 18 Ma have an estimated formation temperature range of between 700 and 800 °C (e.g., typology S9). Grains with ages between 17.0 and 18.0 predominantly show a slightly lower temperature range of between 650 and 700 °C (e.g., typologies P1 and P2), although a few grains show temperatures of up to 850 °C (Fig. 7a). This could suggest the presence of an antecrystic component and/or post-depositional reworking in accordance with the petrologic data. Finally, we note that zircon analyses in a few samples yield U–Pb ages, which are within error significantly younger than the predominant Miocene population (Figs. 4c, 5; Table S2). This in particular holds true for sample CK I from the Crveni Klanac profile, where some grains give ^206^Pb/^238^U ages as young as 15.5 ± 0.3 Ma, much younger than the weighted mean age of 16.9576 ± 0.021 Ma previously obtained by CA–ID–TIMS dating of the youngest Miocene zircons from the same stratigraphic profile (Brlek et al. 2021). Taking this into account, the youngest zircon ages in sample CK I and other samples with ages younger than the main Miocene age peak might be interpreted to result from partial Pb loss. Although, it is worthwhile noting that the U contents of the youngest grains (*U* = 340–550 µg/g) are similar or even lower than those of the grains used to calculate the weighted mean age (*U* = 110–3500 µg/g; see ESM-Table S2). This suggests that Pb loss is unrelated to volume diffusion, controlled and enhanced by radiation damage and metamictizations (Murakami et al. 1991), but rather related to submicroscopic fractures hit during laser ablation.

### Interpretation of the age spectra and calculation of depositional ages

All of the probability density diagrams show a distinctive Miocene age peak (Fig. 5), most likely related to the main eruption that coincided with, or slightly predated, deposition of the respective volcaniclastic layer. The SUT 1 sample shows a double peak, which might indicate that it includes zircons from two temporally closely spaced volcanic eruptions (Fig. 5). Despite these well-defined age peaks, all samples display a spread of Miocene zircon ages, with a fraction being younger and a fraction being older than the main age peak. The younger ages may be explained by Pb loss due to alteration or submicroscopic fractures of the zircon grains (see section Zircon textures). Notably, some grains with multiple analyses display a wide variation of non-overlapping ages, together with a disrupted zoning pattern suggestive of post-growth alteration (e.g., Z829 in Fig. 4c), which may hint at Pb loss. The older ages may be explained by (1) reworking of older volcaniclastic material, (2) antecrystic and/or xenocrystic components.

For the calculation of depositional ages, only samples from group I were selected. These contain the fewest inherited grains, minimizing the risk of including inherited Miocene zircons into the age calculation. We note that some individual ages may represent an average of multiple age domains as the spot analyses sample several zircon zones/bands (Fig. 4). Nevertheless, significant age differences would result in highly discordant analyses, which have been excluded from the calculation. In the case of protracted zircon growth, the range of Miocene ages obtained per sample may be slightly biased towards older values. Therefore, weighted mean ages of the youngest Miocene zircon grains are presented, after rejecting grains which show signs of Pb loss (Fig. 4). This ensures that the weighted mean age captures the youngest zircon crystallization interval, i.e., zircons formed closest to the timing of the eruption. For group II samples, MDAs based on the age of the main Miocene probability density peaks are presented (Fig. 5a; Table 1).

### Age model for the Sinj Basin

The new U–Pb ages obtained from the Sinj Basin, together with previous U–Pb and ^40^Ar/^39^Ar ages, allow stratigraphic correlation of different sections across the basin. The oldest age of ~ 17.7 Ma is provided by the SUT 1 sample of the Lučane section (Figs. 2, 8). This is a maximum depositional age, as the zircon population of sample SUT 1 is dominated by inherited grains (Fig. 5; Table 1). This is in agreement with the reverse polarity of the directly overlying strata (chron C5Dr.1r; Fig. 8), although the lowermost 10 m of the section lack magnetostratigraphic data and could be older (de Leeuw et al. 2010). Taking this into account a maximum age of ~ 17.7 Ma may be proposed for the base of the Lučane section (Fig. 8). At approximately 60 m in the Lučane section (Fig. 8), a volcaniclastic layer which previously yielded an ^40^Ar/^39^Ar age of 17.92 ± 0.18 Ma (de Leeuw et al. 2010) has been re-dated by Brlek et al. (2021), yielding an U–Pb age of 17.312 ± 0.024 Ma. This zircon age fits with the normal polarity in this part of the section, suggesting that it corresponds to chron C5Dn. The previous ^40^Ar/^39^Ar age was obtained on biotites which may provide ages that are slightly older than the depositional age (Villa and Bosio 2022). In the interval between 90 and 135 m, two volcaniclastic layers gave ages of 17.239 ± 0.051 Ma (SUT 3; 90 m) and 17.160 ± 0.052 Ma (SUT 5; 135 m). These ages are slightly older than expected from the polarity pattern (Fig. 8). The slight misfit might perhaps be explained by protracted zircon growth. Significant post-depositional reworking from the basin margins is not likely based on the lack of concordant pre-Miocene zircon grains (Table 1). Some degree of reworking might perhaps only be suggested for SUT 5, based on the mixing of pyroclastic material and characean oogonia (Šegvić et al. 2014). Upwards, the ^40^Ar/^39^Ar age of 16.23 ± 0.16 Ma (de Leeuw et al. 2010) obtained from a volcaniclastic horizon at approximately 233 m provides a good tie-point for correlating the middle part of the section to the ATNTS (Fig. 8). The SUT 6 sample, at 416 m, yielded an age of 15.283 ± 0.034 Ma (Fig. 6). This is slightly younger than the 15.43 ± 0.05 Ma ^40^Ar/^39^Ar age obtained by de Leeuw et al. (2010). The reported ^40^Ar/^39^Ar age was obtained from multiple sanidine grains, which might include inherited components (de Leeuw et al. 2010), perhaps resulting from protracted crystallization. Thus, the large number of analyzed zircon grains in the present study might allow for a better estimation of the eruption age. The probability density plot also shows a symmetrical peak centered at 15.3 Ma, suggesting that the predominant Miocene zircon population used for the age calculation is not significantly skewed by Pb loss or inheritance (compare with sample SUT 1). Moreover, it is pertinent to note that the obtained U–Pb age is in good agreement with the ages of other volcaniclastic horizons across the circum-Pannonian region which presumably represent products of the same eruption (Badurina et al. 2021; Trinajstić et al. 2024). It is also in good agreement with the polarity pattern (Fig. 8) based on which an age of ~ 15.0 Ma has been derived for the top part of the Lučane section (de Leeuw et al. 2010). The LUČ sample from the base of the Lučane-4 subsection yielded a high number of inherited zircons (Fig. 5; Table 1),which is why we abstained from calculating a weighted mean age. While this hinders precise stratigraphic correlation, we note that the main Miocene age peak yields an age of 15.6 Ma. This in agreement with the correlation of the Lučane-4 subsection with the upper part of the Lučane-3 subsection which was previously also concluded from fieldwork (Olujić, 1999; Vranjković et al. 2024). Based on all available data, the lower 290 m of the Lučane section had an average sedimentation rate of approximately 17 cm/kyr (de Leeuw et al. 2010; Brlek et al. 2021; Vranjković et al. 2024). A slight increase of the sedimentation rate to 18 cm/kyr is revealed between 290 and 416 m in the section, corresponding to the base of chron C5Br and the SUT 6 volcaniclastic layer, respectively (Fig. 8). If the top of the Lučane section indeed includes the C5Bn.2n to C5Bn.2r reversal boundary (de Leeuw et al. 2010), then this indicates a marked increase in the sedimentation rate of up to 39 cm/kyr, possibly related to the deposition of coal (Fig. 8). Alternatively, an even higher sedimentation rate may be assumed.The age of the succession in the central part of the basin, represented by the Poljaci and Šolto sections, is calibrated by two U–Pb ages (Fig. 8). A volcaniclastic horizon in the lower part of the Poljaci section yielded an U–Pb age of 17.295 ± 0.028 Ma (Brlek et al. 2023). However, deposition in the central part of the basin likely began earlier, as a few tens of meters of Miocene deposits between the Poljaci section and underlying Permo–Triassic evaporites were not logged due to poor exposure. The ŠOLTO 1 volcaniclastic horizon, near the base of the Šolto section, yielded an age of 16.913 ± 0.043 Ma (this study), which is in agreement with the 16.9567 ± 0.0074 Ma age reported by Brlek et al. (2024b) from approximately the same layer. An average sedimentation rate of 19 cm/kyr can be calculated for this part of the basin infill, using the Poljaci and ŠOLTO 1 volcaniclastic horizons as tie-points.At the Crveni Klanac section, U–Pb–zircon ages obtained from residual deposits constrain the maximum depositional age at 16.9576 ± 0.021 Ma (Brlek et al. 2021). This agrees with the 17.0 and 17.1 Ma main age peaks of the CK I and CK II samples, respectively. A small number of younger analyses in the CK I sample (Fig. 5) likely represents Pb loss. Therefore, zircon populations from the Crveni Klanac bauxite constrain a maximum depositional age of ~ 17.0 Ma for the lacustrine infill of the SE part of the Sinj Basin (Brlek et al. 2021). The predominant Miocene population in the CK 23 sample is meanwhile significantly older, yielding an age peak at 17.4 Ma (Fig. 5). This suggests prolonged accumulation of volcaniclastic bauxite–precursor material, in agreement with the conclusions of Brlek et al. (2021).

Findings of *Illyricocongeria drvarensis* within dreissenid-bearing marls in the Strmendolac area, to the north of Crveni Klanac, (Šušnjara and Sakač, 1988; Jurišić-Polšak et al. 2000) constrain the age of these deposits at between 15.9 and 15.7 Ma, based on biostratigraphic correlation with the Lučane section (de Leeuw et al. 2010; Neubauer et al. 2016). Based on superposition, the progradation of the overlying charophytic limy marls and clayey limestones (Jurišić-Polšak et al. 2000) of the middle unit of the lacustrine infill must have occurred sometime after 15.9 Ma, i.e., not before the first occurrence of *I. drvarensis*.

In conclusion, our new U–Pb ages agree with the previously obtained dating constraints, which demonstrate that deposition lasted from 17.7 to 15.0 Ma (de Leeuw et al. 2010; Brlek et al. 2021, 2023, 2024a, b).

### Detrital zircon populations in the Sinj Basin

**Fig. 9:**
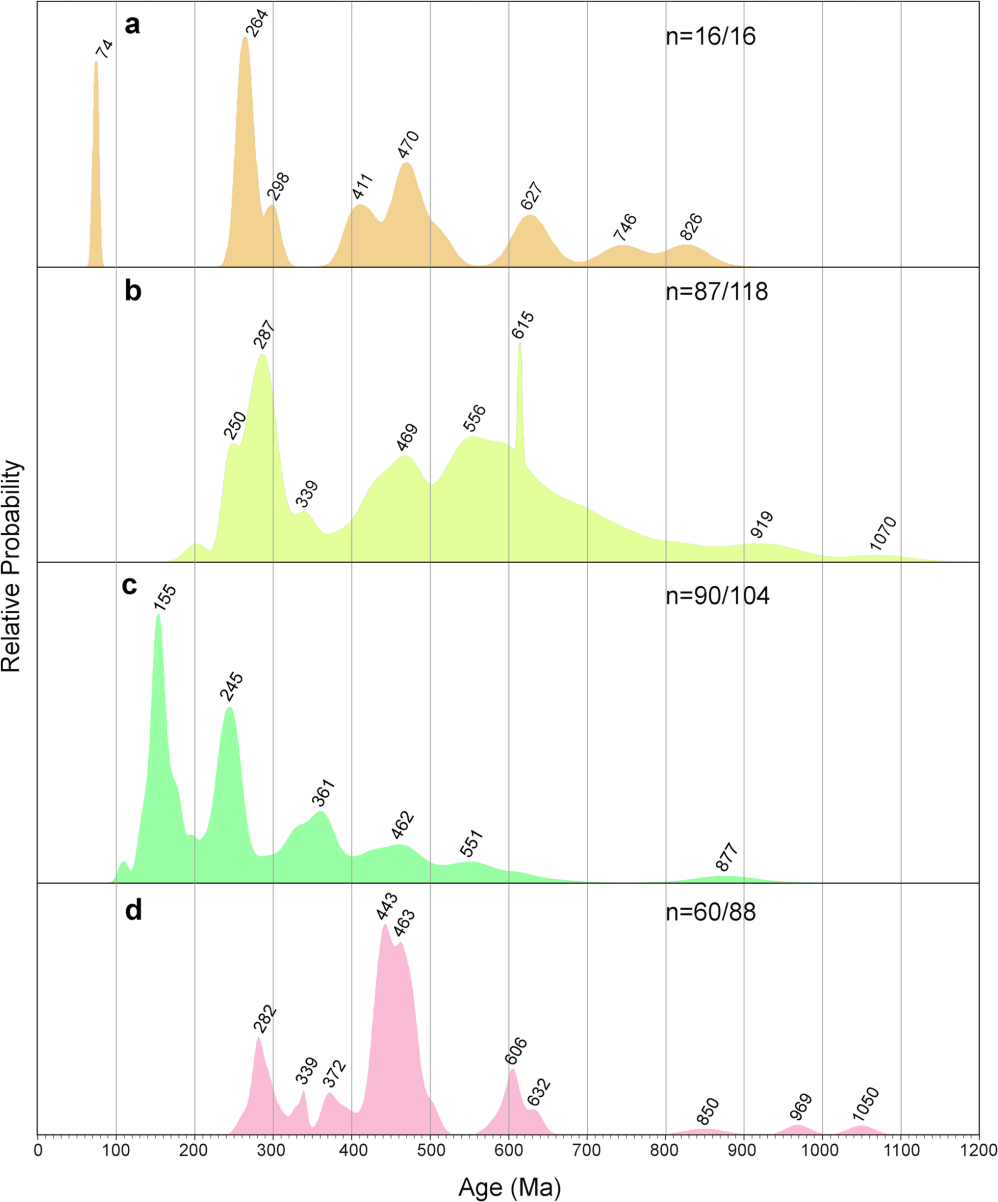
Detrital zircon age spectra of samples from older sedimentary units in the Dinarides. The data are presented in age vs. relative probability diagrams (only data with concordance level between 90% and 110%). **a** Oligocene calcarenite from Mt. Promina (Promina Fm.; sample BXH-4eu; Brčić et al. 2023), **b** Upper Cretaceous Ugar Fm. (sample BO-16; Mikes et al. 2008), **c** Lower Cretaceous Vranduk Fm. (sample BO-12; Mikes et al. 2008), **d** Permo-Triassic siliciclastics of the Bükk Mts. Hungary (Zajzon et al. 2011)

The high amount of detrital zircon grains in all investigated samples of volcaniclastic and residual deposits suggests abundant erosion and redeposition of detritus from older sedimentary units into the Sinj Basin. Most samples show prominent Middle Triassic, Permian, Carboniferous, Devonian, Ordovician, Ediacaran, and Cryogenian detrital zircon age clusters, but there are also minor peaks reflecting detrital zircon input from Oligocene to Early Miocene, Eocene, Early to Late Cretaceous, and Early Jurassic igneous sources (Fig. 5b). Differences in the detrital zircon age spectra between the northwestern, central, and southeastern parts of the basin probably reflect differences in basement lithologies surrounding the basin, but could also result from sampling bias (Fig. 5). Unfortunately, detrital zircon age spectra for pre-Miocene successions surrounding the Sinj Basin are not available, except one sample from the Promina deposits, which was taken ca. 40 km northwest of the study area by Brčić et al. (2023; Fig. 9). Thus, for zircon age-spectra comparison, information from sedimentary successions exposed across the wider Adria area will be used. We note that most of the investigated detrital zircon grains are mononphase, show Th/U ratios > 0.1 (Table S2a), and zoning patterns suggestive of a magmatic origin (e.g., grains Z355, Z427, Z457, and Z1500 in Fig. 4c). Only a few grains show clear core–rim relationships indicative of multiple magmatic growth (e.g., grains Z836, Z713, Z1141, and Z143 in Fig. 4c). In general, the detrital zircon populations can be subdivided into five age groups: (1) pre-Variscan, (2) Variscan, (3) Permo-Triassic, (4) Jurassic–Cretaceous, and (5) Eocene-to-Early Miocene. The oldest age clusters are defined by zircon grains of Neoproterozoic (680–570 Ma), Late Ediacaran (ca. 530 Ma, only found in the samples SUT 4 and LUČ), and Ordovician-to-Early Silurian age (490–440 Ma). Zircon grains of all three clusters are commonly well-rounded (av. DOR 4.0–3.5), reflecting intense reworking through multiple cycles of redeposition and/or metamorphism. Some of these grains show collision and grinding marks at their surfaces (e.g., grains Z355 and Z457 in Fig. 4c), pointing to final zircon transport in littoral and/or aeolian environments (Garzanti et al. 2012, 2015; Zeh and Wilson 2022).

**Fig. 10:**
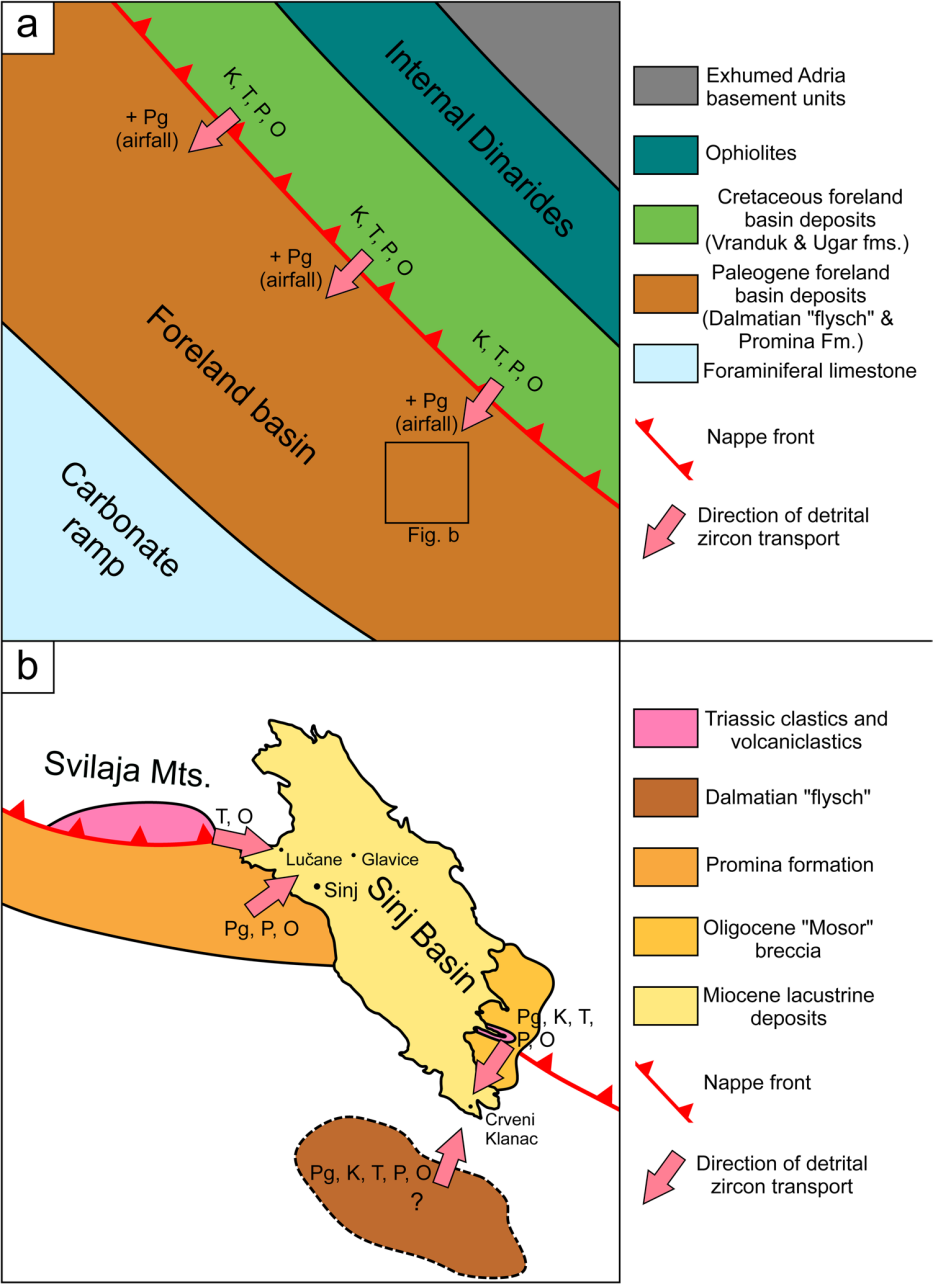
Schematic paleogeographic maps of the study area highlighting the proposed source to sink pathways of detrital zircons during the uplift of the Dinarides. **a** In the Eocene, the uplift of the Internal Dinarides and associated older foreland basin deposits supplied zircons into the Eocene foreland basin of the External Dinarides. In addition, Eocene zircon populations likely entered the basin from tephra air fall. **b** The immediate surroundings of the Sinj Basin during the Miocene, with potential sources and transport direction of zircon detritus admixed within the volcaniclastic layers of the Sinj Basin. *Pg* Paleogene, *K* Cretaceous, *T* Triassic, *P* Permian, *O* Ordovician

The Neoproterozoic zircon population is typical for detritus initially deposited along the northern margin of Gondwana, in the Avalonian–Cadomian Belt (e.g.,Linnemann et al. 2000; Zeh et al. 2001; Kühnemann et al. 2025), whereas the Late Ediacaran to Early Cambrian zircon population was sourced most likely from a continental magmatic arc established along the northern Gondwana margin by this time (e.g., Linnemann et al. 2000, 2007; Stephan et al. 2019). Zircon core–rim relationships suggest magmatic reworking of Neoproterozoic/Cambrian zircon grains during the Devonian at 410 Ma (e.g., grain Z455). The predominant Ordovician–Silurian zircon population, with a prominent peak at 465 Ma, probably was also derived from a magmatic arc system (Stephan et al. 2019), which temporally overlaps with the cryptic Sardic (Cenerian) orogenic phase at 470–450 Ma, reflected by high-pressure metamorphic rocks in the Alps and the Slavonian Mountains (e.g., Zurbriggen 2017; Starijaš-Mayer et al. 2023). We note that a few zircon cores of Ordovician age are surrounded by magmatic rims of Carboniferous (grain Z1141) and Miocene age (e.g., grain Z836 in Fig. 4c, ESM-Table S2), suggesting assimilation of older strata in magma chambers by these times. In the Dinarides, Neoproterozoic, and Early Cambrian zircon grains are known from nearly all investigated siliciclastic rocks, albeit of highly variable amounts (Zajzon et al. 2011; Mikes et al. 2008). Detrital zircon grains of Ordovician age are very common in foreland basin deposits across the Dinarides, including the Upper Cretaceous Ugar Fm. and the Oligocene Promina “molasse” (Fig. 9; Mikes et al. 2008; Brčić et al. 2023). In the Sinj Basin, such grains most frequently occur in samples taken from the NW margin, close to outcrops of lower Triassic sandstones (Fig. 10). We note that abundant detrital zircon grains of Ordovician age were also found in Permo–Triassic sandstones of similar age within the Jadar–Kopaonik thrust sheet in the Bükk Mts. in Hungary (Fig. 9; Zajzon et al. 2011).

The detrital zircon record further provides evidence for widespread magmatism during the Late Devonian–Carboniferous (380–300 Ma), Permian (295–252 Ma), and Triassic (250–220 Ma) with prominent age peaks at 268 and 235 Ma (Fig. 5). The zircon grains formed during all three periods are commonly less rounded compared to pre-Devonian grains. Zircon grains of Devonian–Carboniferous and Triassic ages are least rounded (av. DOR = 2.5), and many of these show perfect euhedral shapes (e.g., grain Z713 and Z276 in Fig. 4c). The Late Devonian–Carboniferous zircon populations reflect magmatism during the Variscan orogeny (Stephan et al. 2019), and the Early Permian populations reflect volcanic activity, related to post-orogenic collapse, mantle upwelling, and peneplainization following the Variscan orogeny (e.g., Wilson et al. 2004 and references therein). Late Permian and Triassic populations most likely formed by magmatic activity related to the opening of the Neotethys in the Alpine Realm (e.g.,Szoldán 1990; Harangi et al. 1996; Dunkl et al. 2019), including the widespread Middle Triassic syn-rift volcanism of the Adriatic plate (Smirčić et al. 2018). Although some geochemical data point to a magmatic arc setting by this time (e.g.,Bébien et al. 1978; Beltrán-Triviño et al. 2016 and references therein). The angular shape of most Triassic zircon grains in the Sinj Basin suggests a proximal source, perhaps the Middle Triassic volcaniclastic deposits exposed in the southern Svilaja Mts. (Smirčić et al. 2018) close to the Sinj basin, or the Dinaric foreland basin deposits (Mikes et al. 2008). Jurassic and Cretaceous zircon grains are very rare in the investigated samples but deserve special attention with respect to the tectono–sedimentary evolution of the Dinarides. Only three grains were found during this study, showing Early Jurassic (ca. 196 Ma; CKM 23), Early Cretaceous (138 Ma; CK I), and Late Cretaceous ages (84 Ma; CK I). We note that evidence for Jurassic magmatism is very rare in the Dinarides, albeit Early Jurassic zircon grains of detrital origin were commonly found in foreland basin deposits of both the Internal and External Dinarides (Mikes et al. 2008; Löwe et al. 2023). The Early Jurassic zircon in the Sinj Basin sample perhaps resulted from volcanic activity related to rifting of the Alpine Tethys (van Hinsbergen et al. 2020), whereas the nature of the Early Cretaceous grain is ambiguous. This grain shows a round shape and a disturbed internal zoning (grain Z31 in Fig. 4c), which might reflect zircon alteration accompanied by age reset, perhaps related to Late Jurassic–Early Cretaceous ophiolite obduction in the Internal Dinarides (Mikes et al. 2008). In contrast, the Late Cretaceous zircon (84 Ma) shows banded internal zoning and a perfect euhedral shape (Fig. 4c; grain Z41), suggesting a pristine habitus and supply from a proximal source, perhaps from the Late Cretaceous Apuseni–Banat–Timok–Srednogorie magmatic arc, which developed on the European margin during the closure of the Neotethys (Gallhofer et al. 2015). Volcanic zircons originating from this magmatic arc have been reported from Upper Cretaceous foreland basin successions of the Internal Dinarides (Stojadinović et al. 2017). The youngest detrital zircon grains of igneous origin in the Sinj Basin show an Eocene age (42 Ma), obtained from sample LUČ (Fig. 5). A middle Eocene population has already been reported from bauxites of northern Dalmatia (Brčić et al. 2023), perhaps sourced from volcanic centers located either within the Internal Dinarides or the Alps (Cvetković et al. 2013; Ji et al. 2019). This period of volcanic activity overlaps with deposition of the Dalmatian “flysch” and the Promina deposits (Babić et al. 1995; Ćosović et al. 2018; Mrinjek et al. 2012; Papeš et al. 1982), which both represent the most likely intermediate sources of the Eocene zircon grains found within the Sinj Basin. Inherited zircon grains of late Oligocene–early Miocene ages were already noted in the Crveni Klanac area by Brlek et al. (2021). Presently, no coeval deposits are known from the Sinj basin margins, except possibly the Mosor breccia. Tephra transport of at least some of the inherited zircon domains is documented by grain Z143 (Fig. 4e), which shows a CL-bright inherited core of Oligocene age (33 Ma) surrounded by a magmatic rim of Miocene age (16.7 Ma). This is in agreement with the Oligocene zircon population reported by Brlek et al. (2021) from the Crveni Klanac section. Our results highlight that at least part of these Oligocene ages might represent products of an earlier phase of magmatic activity in the Pannonian Basin, which were assimilated and emplaced together during the subsequent Miocene volcanism.

### Provenance of detrital zircon grains

The combined information of zircon ages and morphologies suggests that most of the detrital zircon grains went through multiple cycles of erosion, transport, and deposition, in particular the zircon grains of pre-Variscan and Variscan age, but also the Mesozoic zircon grains. From field relationships it is likely that most of the detritus in the Miocene volcaniclastic layers of the Sinj Basin stems from two major intermediate sources: (1) from reworked Triassic strata of the adjacent Svilaja Mts. as already postulated by Šušnjara and Šćavničar (1974) and (2) from the Eocene–Oligocene foreland basin deposits, including the Promina “molasse” (Brčić et al. 2023). The first interpretation is backed by the research of Środoń et al. (2018), showing that exhumation of Triassic strata of the Svilaja Mts mainly occurred during the Eocene–Oligocene, as well as by palynomorph data of Jiménez-Moreno et al. (2008), suggesting an elevated mountain topography around the Sinj Basin during the Miocene. Nevertheless, the presence of post-Triassic detrital zircons in the Sinj Basin necessitates supply of detrital material also from the surrounding foreland basin deposits. These include the Eocene Dalmatian “flysch,” late Eocene–Oligocene Promina “molasse,” and the Oligocene Mosor breccia (Fig. 10). As the Eocene “flysch” of the External Dinarides was at least partly sourced from the uplift and erosion of the Internal Dinarides and their foreland basin deposits (Tari 2002; Mikes et al. 2006), it is likely that post-Triassic detrital zircon populations were recycled first into the Eocene Dalmatian “flysch,” and subsequently into the Sinj Basin (Fig. 10). The closest present-day exposures of the Eocene Dalmatian “flysch” occur ca. 10 km to the south of the Crveni Klanac section (Marinčić et al. 1969). This implies that Eocene siliciclastics possibly covered a wider area around the southern part of the Sinj Basin during the Miocene. An alternative intermediate source for the post-Triassic detrital zircon populations in the Sinj Basin could have been the Oligocene Mosor breccia or the late Eocene–Oligocene Promina “molasse.” The latter contains Late Cretaceous zircon grains (Fig. 9; Brčić et al. 2023) and lithoclasts of older foreland basin deposits (Gobo et al. 2024), suggesting multiple recycling of foreland basin deposits during the uplift of the External Dinarides. A direct recycling of Cretaceous foreland basin deposits into the Sinj Basin is unlikely, as these occur within structurally more internal units (e.g. Mikes et al. 2008), which were separated from the Sinj Basin by high topography (Brlek et al. 2024a; Jiménez-Moreno and Mandic 2020). Regarding the source areas of the Eocene–Oligocene foreland basin deposits, it is worth noting that while Middle Jurassic zircon populations are common in the Vranduk Fm. (Fig. 9, Mikes et al. 2008) they are absent in the Sinj Basin. Therefore, recycling of the Vranduk Fm. may be excluded. The younger Ugar Fm. remains a viable source (Fig. 9), as well as “flysch” deposits of the High-Karst nappe of which presently only a few erosional remnants remain (Velić et al. 1979). Additional recycling of various Paleozoic zircon populations from exhumed Paleozoic deposits in the Internal Dinarides (Hrvatović 2022), or from the Tisza–Dacia unit, into the foreland basin deposits of the External Dinarides is also a viable option (cf. Ustaszewski et al. 2010).

### Tectonic implications

In summary, the detrital zircon populations of the Sinj Basin volcaniclastic layers reflect three major tectono-volcanic processes related to the Dinaric orogeny: (1) Cretaceous–Eocene uplift and erosion of the Internal Dinarides and their foreland basin deposits causing the supply of Cretaceous, Triassic, Permian, Carboniferous, and possibly also Ordovician and Neoproterozoic zircon populations into younger foreland basin deposits of the External Dinarides, accompanied by Eocene tephra zircon input from coeval volcanic sources (Fig. 10a). (2) late Eocene–Oligocene uplift of the External Dinarides (Korbar 2009; Mrinjek et al. 2012) initiated the erosion of the foreland basin deposits. These supplied detritus into the Sinj Basin during the Miocene. (3) Erosion of Triassic strata in the adjacent Svilaja Mts. probably provided an additional source for the predominant Triassic and Ordovician zircon populations found in the Lučane section (Fig. 10b). If the Triassic deposits of the Svilaja Mts. were indeed exhumed to the surface by the time of Early Miocene deposition in the Sinj Basin, then most of the thrusting along this segment of the Split–Karlovac fault must have occurred prior to the Miocene, in line with the interpretation of Balling et al. (2021). Accordingly, the possible post-Middle Miocene transpression (Neubauer et al. 2016) must have produced relatively minor exhumation and uplift.

### Paleogeographic evolution of Lake Sinj

**Fig. 11:**
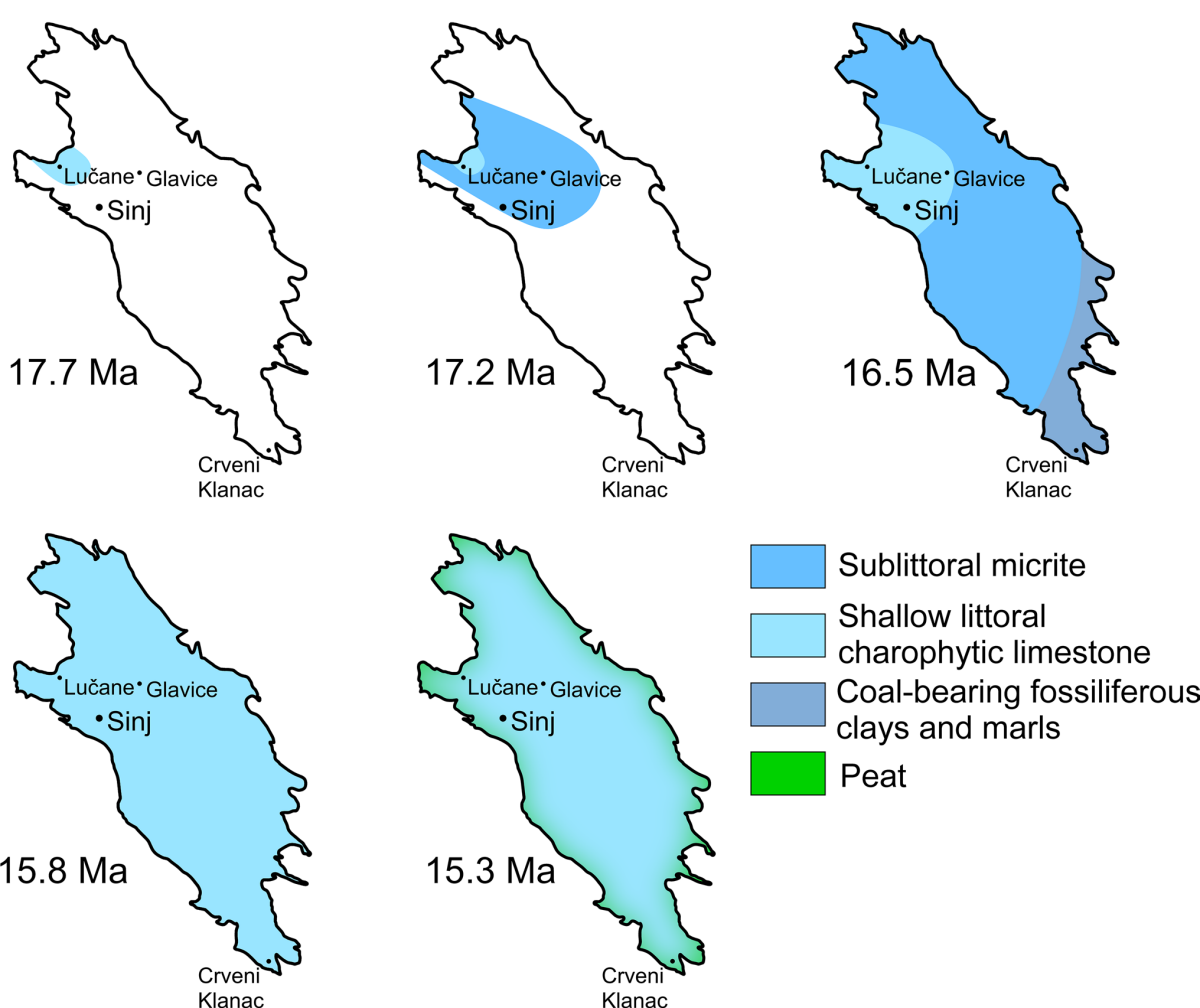
Simplified paleogeographic maps showing the depositional evolution of Lake Sinj, superimposed over the present-day basin outline (for details see chapter Paleogeographic evolution of Lake Sinj)

The results of our U–Pb dating, integrated with results from previous publications (de Leeuw et al. 2010; Brlek et al. 2021; 2024a, b) suggest that deposition in different parts of the Sinj Basin was diachronous. Lacustrine flooding commenced in the western part of the Sinj Basin at ~ 17.7 Ma (Fig. 11), as indicated by the magnetostratigraphy and available dating constraints (de Leeuw et al. 2010; Brlek et al. 2021). The lake was initially confined to the western part of the basin, and by ~ 17.3 Ma expanded towards the middle part as indicated by the volcaniclastic layer at the Poljaci section (Brlek et al. 2023). Although the lake was probably small, deposition occurred in a relatively deeper lacustrine environment, implied by the predominance of lime mudstone (Vranjković et al. 2024). Coarse breccias consisting of Mesozoic and Paleogene carbonates (Vranjković et al. 2024) were periodically deposited in the northwestern part of the basin between 17.5 and 17.2 Ma (Figs. 2, 9). Lacustrine flooding reached the Crveni Klanac area at the SE basin edge after ~ 17.0 Ma. The shallow bench platform (Vranjković et al. 2024) started prograding from the Lučane area between ~ 17.2 and ~ 17.1 Ma, and reached the Šolto section area in the central part of the basin after 16.7 Ma, suggested by the 16.913 ± 0.043 Ma age of the ŠOLTO 1 sample and assuming a sedimentation rate of 19 cm/kyr (Figs. 2, 6). Syn-sedimentary diapirism of the Permo–Triassic evaporites presently exposed in the central part of the basin (Fig. 1) is unlikely based on the similar subsidence rates between the Lučane and Glavice sections, and the lack of reworked older lake carbonates in the Glavice section. Meanwhile, in the SE clays and dreissenid-bearing marls with coal intercalations were being deposited (Fig. 11; Šušnjara and Sakač, 1988; Jurišić-Polšak et al. 2000). The paleontological and lithological characteristics suggest a shallow lacustrine environment with significant terrigenous influx, in contrast to the Lučane section. The dreissenid-bearing marls pass upwards into limy marls and clayey limestones with ostracod and characean remains (Jurišić-Polšak et al. 2000), likely corresponding to the charophytic micrite facies (Vranjković et al. 2024). The finding of *Illyricocongeria drvarensis* (Šušnjara and Sakač, 1988) suggests that progradation of this facies occurred after 15.9 Ma in the southwestern part of the basin. The lower part of the Sinj Basin infill thus records a variety of depositional facies, which were likely influenced by the lithology of surrounding bedrock. Moreover, variations of basin morphology probably played a role, either due to differential subsidence, which based on the available data cannot be completely ruled out, or due to variations in pre-depositional paleorelief, as indicated by the ~ 700 kyr minimum timespan before lacustrine flooding reached the SE part of the basin (Figs. 8, 9). After around 15.9 Ma, most of the basin was dominated by a shallow-littoral environment characterized by deposition of the charophytic micrite facies. This is shown by the areal extent of the middle unit of the lacustrine infill (Fig. 1), as well as the occurrence of the aforementioned facies towards the top of the Lučane, Glavice and Crveni Klanac sections (Fig. 8). Although the subsidence rate increased, the facies uniformity of the middle part of the Sinj Basin infill implies a stabilization of the depositional environment and a balance between subsidence and deposition rates. The relatively cooler climate after 16.4 Ma (Methner et al. 2020) was possibly favorable for stable, shallow-littoral charophytic micrite deposition. Coal intercalations appear after ~ 15.3 Ma in the upper unit of the basin infill at the Lučane section. These have been interpreted to mark the gradual shallowing of the lake (Vranjković et al. 2024). This correlates with a warming trend towards the end of the MCO (Methner et al. 2020). Formation of the coals has been related to an increasing abundance of aquatic vegetation, forming peats which prograded from the basin margins (Fig. 11), culminating with the establishment of fen or bog environments within the former lake (Mandic et al. 2009; Vranjković et al. 2024). This likely contributed to a rapid filling of the basin, suggested by the high sedimentation rates in the uppermost part of the Lučane section. Moreover, palynomorph data link coal formation to relatively drier periods (Jiménez-Moreno et al. 2008), possibly reflecting temporary decreases of the lake level. While it is impossible to precisely correlate the intraclastic conglomerates of the Lučane-4 subsection with the coal intercalations at the Lučane-3 subsection, both types of deposits possibly resulted from considerable lake-level falls (Vranjković et al. 2024), with the lake margin being exposed and subjected to erosion, whereas relatively more distal areas were characterized by the progradation of swamps. Increasingly frequent coal intercalations mark the end of the basin's depositional evolution, likely around 15 Ma (Fig. 8). This approximately coincides with the second peak of the MCO (Methner et al. 2020), and highlights the key influence of paleotemperature variations on lake development.

### Geodynamic constraints on the evolution of lake systems in the Dinarides–Carpathian region

**Fig. 12:**
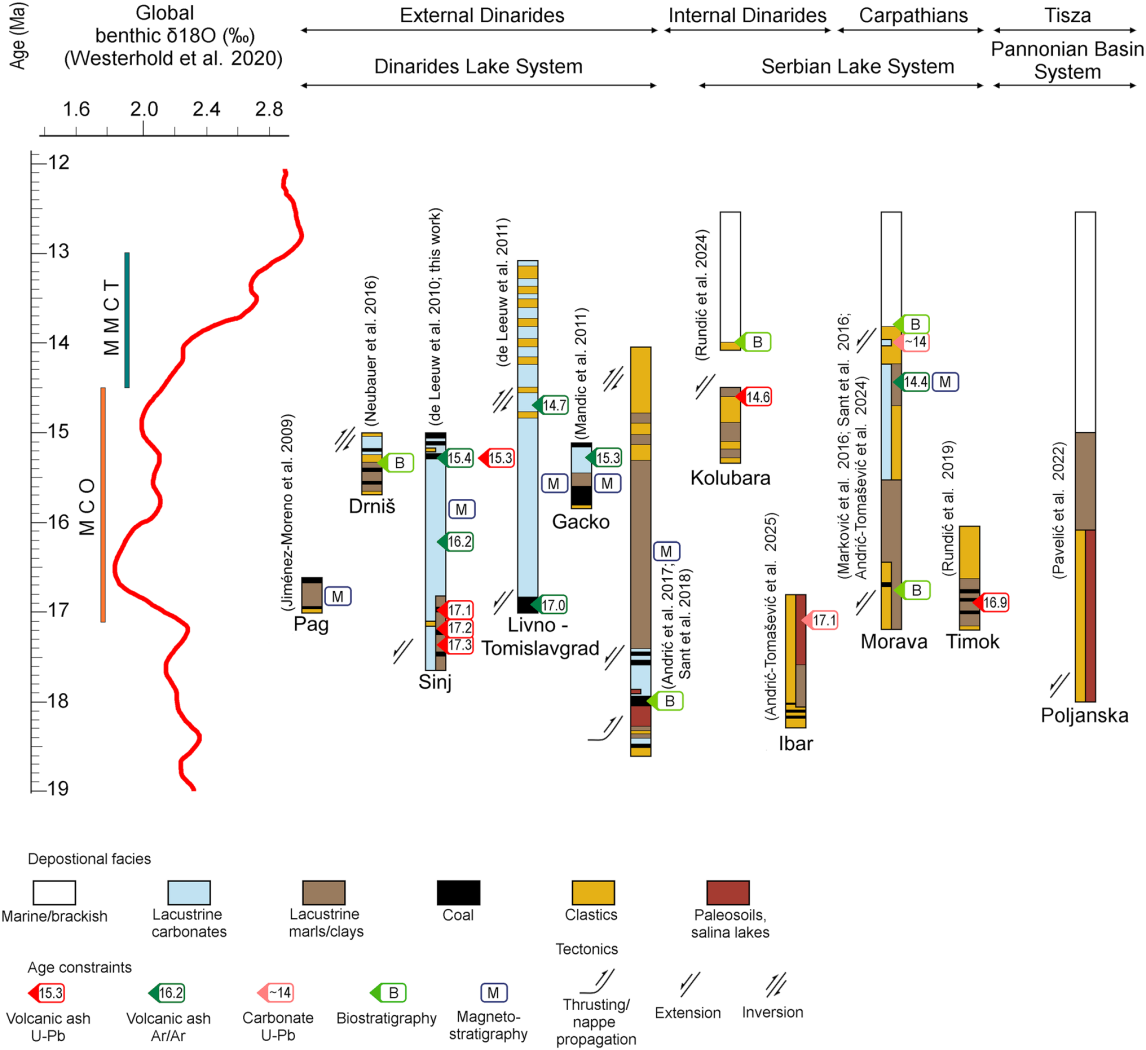
Chronostratigraphic correlation of the Sinj Basin lacustrine succession with the global δ^18^O curve and depositional environments and tectonic events recorded across other Miocene lacustrine basins of the Dinarides and Serbian lake systems. *MCO* Miocene Climatic Optimum, *MMCT* Middle Miocene Climatic Transition

During the Early to Middle Miocene, a profound episode of basin formation affected the Dinarides and neighboring areas, giving rise to the Dinarides and Serbian Lake Systems. Most researchers agree that lake basin development was governed by a complex interplay of extensional tectonics and favorable climatic conditions (Andrić et al. 2017; de Leeuw et al. 2011; 2012; Sant et al. 2016; 2018; van Unen et al. 2019a), although the exact mechanisms remain poorly constrained. The Oligo–Miocene extensional tectonics in the Dinarides have been alternatively attributed to either (1) far-field effects of the Carpathian (Andrić et al. 2017; de Leeuw et al. 2011, 2012) and/or Hellenic slab rollbacks (Handy et al. 2019), (2) eduction during and after slab detachment in the Dinarides (Andrić et al. 2018), or (3) to the rollback of a Dinaric slab (Matenco and Radivojević, 2012; Schefer et al. 2011). Extension related to the Carpathian slab-rollback started in the Early Miocene and peaked from the late Burdigalian until the end of the Serravallian, roughly between ~17 and ~12 Ma (Balázs et al. 2016; Horvath et al. 2015). However, extension and core complex formation along the Sava suture zone commenced already in the late Oligocene (Stojadinović et al. 2017; Ustaszewski et al. 2010; Toljić et al. 2013), requiring the involvement of a Dinaric slab. The minor deformations of Oligo-Miocene strata along the orogenic front of the Dinarides (Fantoni and Franciosi 2010) seem to favor slab detachment as the initial cause of extension along the Sava suture zone (Andrić et al. 2018), followed by more widespread Miocene extension driven by the combined effects of the Carpathian and/or Hellenic slab rollbacks (Handy et al. 2019). Recent developments in chronostratigraphic dating of the Miocene intramontane basins across the Dinarides enable the correlation of major tectonic events and paleoenvironmental conditions (Fig. 12). In the Internal Dinarides, the transition from the preceding Oligocene–Early Miocene NE–SW directed compression (Ilić and Neubauer 2005) to an extensional tectonic regime can be observed in the Sarajevo–Zenica Basin (Fig. 12). This is characterized by a change from foredeep settings towards syn-kinematic lacustrine deposition atop NE-dipping listric faults (Andrić et al. 2017), shown to have occurred between 18.5 and 17.5 Ma (Sant et al. 2018). Correlation of intramontane basin deposits (Fig. 12) shows that by 17 Ma extension affected a broad area ranging from the External Dinarides in the west to the Carpathians in the east, as exemplified by the onset of deposition in the Livno–Tomislavgrad Basin, where an extensional setting has been inferred based on the relatively high subsidence rates (de Leeuw et al. 2011), and the onset of deposition in the Morava graben, where the oldest exposed syn-extensional terrestrial clastics have been biostratigraphically dated at between 17.2 and 16.6 Ma (Marković et al. 2016).

Several hypotheses have been proposed for the origin of the Sinj Basin. A pull-apart tectonic setting (Mandic et al. 2009) can be discounted based on the relatively low subsidence rate of ~20 cm/kyr compared to over 100 cm/kyr for pull-apart basins, such as the Fohnsdorf Basin in the Alps (Sachsenhofer et al. 2000), or the Dead Sea rift (ten Brink and Flores 2012). An extensional setting is plausible given the widespread extension within the Dinarides during the Middle Miocene (van Unen et al. 2019a). This extension is interpreted to have been mostly accommodated within rheologically weak Cretaceous “flysch” deposits along major thrust contacts (Andrić et al. 2017; van Unen et al. 2019b). It is possible that the Split–Karlovac fault was reactivated in a similar manner during this time, as it is developed along Permo–Triassic evaporites which acted as a major decollement surface during the previous Paleogene contraction (Balling et al. 2021). Thermal modeling of the Poštak–Knin block, situated along the aforementioned fault some 40 km to the NW of the Sinj Basin, suggests that it was tectonically active in the Miocene (Środon et al. 2018). Likewise, the deposition of a coarse breccia in the central part of the Sinj Basin, at the top of the Poljaci section, as well as breccia lenses at the Lučane–1 subsection (Figs. 2, 8), possibly indicate short tectonic pulses in the area of the Sinj Basin between ~17.5 and ~17.2 Ma. As carbonates mostly weather through chemical dissolution, a different process is required to produce such deposits. In the Dinarides, recent carbonate rockfall breccias and offshore mass-transport deposits are mostly related to strong seismicity within areas undergoing neotectonic deformation, e.g., Vipava Valley (Popit et al. 2017), Kvarner Gulf (Korbar et al. 2020; Benac et al. 2023), Vinodol Valley (Jagodnik et al. 2020; Palenik et al. 2019), and the coast of southern Montenegro (Biermanns et al. 2022). van Unen et al. (2019b) observed syn-depositional normal faults in the Miocene strata of the Sinj Basin. In addition, Lake Sinj remained hydrologically open (Mandic et al. 2009; Šegvić et al. 2014), and its sediments accumulated in the photic zone, suggesting active subsidence during its formation. It has been suggested that the Sinj Basin developed in response to the dissolution of Permo–Triassic evaporites and collapse of overlying strata during the humid conditions of the MCO (Vranjković et al. 2024). However, similar subsidence rates between the Lučane and Glavice sections rule out significant diapirism during this time (Fig. 8). We also note that the majority of recent evaporite karst lakes and associated geomorphologic features are rather small and short-lived compared to Lake Sinj (Doğan and Özel 2005; Gökkaya et al. 2021; Gökkaya and Gutiérrez 2022; Morellón et al. 2009; Nicod 2006; Valero-Garcés et al. 2014). While dissolution of the evaporite complex could have increased the salinity of the lake (e.g., Levy et al. 2018), this may not be the case with hydrologically open lakes (Nicod 2006). Taken all together, this suggests that extensional tectonics played a key role in the formation of the Sinj Basin, as suggested by de Leeuw et al. (2012), but karstification leading to the formation of a large polje which was subsequently flooded through increased precipitation may have played an additional role (Vranjković et al. 2024). The demise of Lake Sinj at 15 Ma is marked by increasingly frequent coal intercalations and coincides with the peak of the MCO (Fig. 12). However, coarse clastic deposits occurring in several DLS basins after ~ 15 Ma could also mark a tectonically induced end of lacustrine deposition (Fig. 12; de Leeuw et al. 2011, 2012; Neubauer et al. 2016; Andrić et al. 2017; Sant et al. 2018). This Mid to Late Miocene compressional episode has been linked to the continuing northward movement and indentation of Adria, leading to N–S shortening and wrench tectonics in the Dinarides (Ilić and Neubauer 2005; van Unen et al. 2019b; Bjelogrlić et al. 2026). The Split–Karlovac fault was possibly reactivated during this time, leading to the partitioning of the Sinj and Drniš basins by southward thrusting of the Svilaja Mts. block (Neubauer et al. 2016). This is corroborated by AMS data which suggest widespread post-Middle Miocene shortening along the frontal part of the Dinarides (de Leeuw et al. 2012). Therefore, compression in the External Dinarides related to the indentation and counterclockwise rotation of Adria possibly already started at ~ 15 Ma, reaching the Internal Dinarides by the Late Miocene (8–10 Ma, Ibar Basin, Andrić et al. 2015).

Meanwhile, the onset of a new phase of basin development in the Serbian Lake System occurred between 15 and 14 Ma (Sant et al. 2016; Rundić et al. 2024; Fig. 12). Extension in the Internal Dinarides was likely related to effects of the Carpathian slab rollback as it coincided with the peak of extension in the Pannonian basin (Balázs et al. 2016; Matenco and Radivojević, 2012; Sant et al. 2016). Moreover, recent paleomagnetic results indicate significant oroclinal bending of the Sava suture zone during the Early to Middle Miocene, particularly in the area of the Serbian Lake System, driven by the retreating Carpathian slab (Marton et al. 2021; Stojadinović et al. 2022a). Syn-depositional extension is documented in the Morava graben at ~ 14 Ma by tectonically induced travertine deposition near normal faults (Andrić-Tomašević et al. 2024).

### Paleoclimatic implications

In addition to tectonics, correlation of intramontane deposits with the global δ^18^O benthic foraminifera record (Westerhold et al. 2020; Fig. 12) shows that climate played a major role in lake development. The widespread deposition of coal within the Dinarides and Serbian Lake Systems (de Leeuw et al. 2011; Marković et al. 2016; Rundić et al. 2019; Sant et al. 2018) suggests warm and humid conditions prevailed throughout the Dinarides between ~ 17 and ~ 16.5 Ma, coinciding with the onset of the MCO. It is possible that the development of smaller, shorter-lived intramontane basins such as lakes Pag, Drniš, and Gacko was primarily governed by climate as opposed to tectonics. Lake Pag developed during the warming trend at the onset of the MCO (Jiménez-Moreno et al. 2009), whereas lakes Drniš and Gacko formed during a relatively cooler period which commenced after 16 Ma (Mandic et al. 2011; Neubauer et al. 2016). On the other hand, deposition in these basins was likewise terminated during a warming trend which started after 15.3 Ma, and which also coincides with the shallowing of Lake Sinj (Fig. 12). Meanwhile, the longevity of the larger lakes such as Livno–Tomislavgrad and Sarajevo–Zenica appears to have been governed by sustained tectonic subsidence.

During this time frame, the lacustrine systems occupying the Dinarides and their transition towards the Pannonian Basin recorded contrasting depositional conditions. Namely, sedimentological and paleontological data imply humid climatic conditions along the western flank of the Dinarides, while the eastern flank was affected by dry climatic conditions (Internal Dinarides). This is inferred from the finding of Mg-bearing clays and oil shales in the Miocene Pranjani Basin which point to a saline lake environment with a stratified water column (Andrić-Tomašević et al. 2021). These authors suggested that the growth of the Dinarides was responsible for such contrasting climatic conditions by creating a rain shadow effect and blocking the moisture coming from the Adriatic Sea. Regional aridity is also suggested from Early Miocene saline-alkaline lacustrine successions along the southern Pannonian Basin (Pavelić et al. 2022; Burazer et al. 2025). In addition, U–Pb calcite dating of borate-bearing salina lake deposits from the Ibar Basin, yielding an age of 17.13 ± 0.43 Ma (Andrić-Tomašević et al. 2025), is consistent with coeval contrasting climatic conditions in the Dinarides (Fig. 12).

Our detrital zircon ages, and estimates of exhumation rates in the External Dinarides (Środoń et al. 2018), are in agreement with major uplift and erosion prior to the formation of the Miocene lake basins. Kocsis et al. (2014) estimate 1200 ± 340 m of uplift along the Alps–Dinarides chain after 31 Ma, based on δ^18^O fractionation between the western part of the Alpine Molasse Basin and Pannonian Basin. Further sedimentological and geochronological constraints are needed, especially from the deposits of the Serbian Lake System, to asses the presence of an east–west climatic gradient across the Dinarides during the Early to Middle Miocene and its relationship with the global climate and timing of regional uplift. In particular, recent studies on the provenance of the volcaniclastic material deposited within the lacustrine successions of the DLS suggested the silicic volcanism of Pannonian–Carpathian region as the likely source (Badurina et al. 2021; Brlek et al. 2023, 2024b), implying dominant easterly trade winds in the area of the Dinarides during the Middle Miocene. Our estimates of eruption temperatures based on zircon morphology (Pupin 1980), which range between ~ 700 and ~ 830 ℃ (Fig. 3) are consistent with this interpretation. However, this is challenged by climate modeling suggesting a westerlies-dominated wind-field in Southeastern Europe during the Middle Miocene, and predicting decreasing levels of precipitation across the Dinarides towards the east (Botsyun et al. 2022). This is consistent with δ^18^O and δ^13^C data which suggest the dominance of westerly-driven moisture transport across Europe already in the early Oligocene (Kocsis et al. 2014).

## Conclusions

1. Zircon roundness and detrital zircon content may be useful for disriminating the degree of post-depositional reworking of volcaniclastic layers. Group I samples (SUT 3, SUT 5, SUT 6 and ŠOLTO 1) with mostly angular magmatic zircon grains and ≤ 6% of inherited grains were minimally reworked and serve as reliable markers for the timing of deposition. Group II samples (SUT 1, SUT 4, ŠOLTO 2, CK I, CK II and CKM 23) represent mixed silici- and volcaniclastic material and contain relatively more rounded grains, and > 50% inherited zircons. In such samples, the youngest zircon population represents a maximum depositional age, whereas the older populations mostly reflect recycling of sedimentary detritus from the basin margins.
2. Our new U–Pb ages agree with the established chronostratigraphy of the Sinj Basin, showing that deposition occurred between ~ 17.7 and ~ 15.0 Ma.
3. Integration of the new U–Pb data with existing age constraints provides a review of the diachronous deposition in the Sinj Basin. Lacustrine flooding progressed from the northwest towards the southeast, commencing in the Lučane section after 17.7 Ma, reaching the central part by 17.3 Ma, and the SE basin margin after 17.0 Ma. A shallow-littoral carbonate bench platform started prograding from the NW to the SE after 17.2 Ma.
4. After 15.9 Ma, the entire basin was affected by stable, shallow water carbonate sedimentation, coinciding with a period of global cooling, whereas frequent coal intercalations after 15.3 Ma indicate gradual shallowing of the basin related to a period of global warming.
5. Deposition in the basin ceased at the peak of the MCO.
6. The overall stable subsidence rates of ca. 17–19 cm/kyr and relatively shallow depositional environments preclude significant syn-sedimentary diapirism and suggest that basin development was driven by continuous subsidence within an extensional tectonic regime.
7. Detrital zircon grains in the Sinj Basin volcaniclastic and residual deposits reveal a wide spectrum of ages from the Neoproterozoic to the Early Miocene, whereby the youngest inherited grains may account for minor inconsistencies between calculated depositional ages and magnetostratigraphic constraints.
8. Detrital zircon ages and morphologies suggest multiple recycling of silici- and volcaniclastic material, first by Cadomian to Variscan orogenic and erosional events, second by the Cretaceous–Eocene uplift of the Internal Dinarides, second by late Eocene–Oligocene uplift of the External Dinarides and their foreland basin deposits, and third by erosion of the foreland basin deposits and the tectonically exhumed Triassic units in the Svilaja Mts., and their mixing with younger tephra deposits during the Miocene lacustrine sedimentation in the Sinj Basin.
9. Correlation of the stratigraphy of Miocene intramontane basins in the Dinarides shows a complex relationship between climate and tectonics, including several extensional episodes. Lakes on the western side of the mountain range (DLS) are characterized by carbonate deposition within a humid climate. In contrast, lakes on the eastern flank (SLS) formed within relatively arid environments, highlighting the need for better age constraints to constrain the duration and extent of this east–west climatic gradient in the Dinarides during the Miocene.

## Supplementary Information

Below is the link to the electronic supplementary material.

Supplementary file1 (DOCX 22 KB)

Supplementary file2 (XLSX 76015 KB)

Supplementary file3 (XLSX 48 KB)
