# Supplementary material for: U–Pb dating of volcaniclastic deposits from the Sinj Basin: implications for provenance and the tectono-sedimentary evolution of the External Dinarides
Source: Int J Earth Sci. 2026 Apr 11;115(2):27. doi: 10.1007/s00531-026-02567-w (PMC13070079; doi:10.1007/s00531-026-02567-w)
Supplement: Supplementary file 1 — Supplementary file1 (DOCX 22 KB) [file 531_2026_2567_MOESM1_ESM.docx]

Table S1: Operating conditions for the LA-ICP-SF-MS equipment for U-Th-Pb isotope analysis

| **Laboratory & Sample Preparation** |  |
| --- | --- |
| Laboratory name | Institute for Applied Geosciences, KIT (Karlsruhe Institute of Technology), Germany |
| Sample type/mineral | tephra zircons |
| Sample preparation | Jaw crusher, disc mill, panning, conventional mineral separation, 1 inch resin mount, polish to finish (all at KIT) |
| Imaging | BSE-images: VEGA TESCAN with Oxford detector, BSE mode 15 kV, <10nA (at institute for applied geosciences, KIT)  CL images: Thermofisher Scientific Quattro S Environmental Scanning Electron Microscope (ESEM), 15kV (at institute for functional interfaces, KIT) |
| **Laser ablation system** |  |
| Make, Model & type | Teledyne Photon Machines, Analyte Exite+ (Excimer) |
| Ablation cell | Two-volume ablation cell (HELEX 100, EQC COMP), ANUAustralia) |
| Laser wavelength | 193 nm |
| Pulse width | < 5 ns |
| Fluence | 2.0 J/cm^-2^ |
| Repetition rate | 10 Hz |
| Spot size | 20 µm (session 1 & 2); 35 µm (session 3); 65 µm (ref. zircon SING low-U) |
| Sampling mode / pattern | single spot |
| Carrier gas | He(cell) 0.30 l/min, He (cup) 0.20 l/min, 0.94-0.96 l/min Ar, N_2_ (10-12 ml) |
| Background collection | 15 seconds |
| Ablation duration | 15 seconds |
| Pre-ablation | 3 pulses |
| Wash-out delay | 15 seconds |
| Cell carrier gas flow (He) | 0.50 l/min (total) |
| **ICP-MS Instrument** |  |
| Make, Model & type | Thermo-Scientific ELEMENT XR (sector field) |
| Sample introduction | via conventional tubing |
| RF power | 1240 W |
| Sampler, skimmer cones | Ni-Jet-cone |
| Extraction lenses | X - type |
| Make-up gas flow (Ar) | 0.94-0.96 l/min |
| Detection system | single collector secondary electron multiplier |
| Data acquisition protocol | Time-resolved analysis |
| Scanning mode | Peak hopping, four point per peak |
| Detector mode | Pulse counting mode |
| Masses measured | ^202^Hg,^204^(Hg + Pb), ^206^Pb, ^207^Pb, ^208^Pb, ^232^Th, ^238^U |
| Integration time per peak | 10 milliseconds |
| Sensitivity / Efficiency | 26000-32000 (cts/ppm) (^238^U, NIST612, spot-20μm, 10μm/s, 10 Hz, 5.0 J/cm^2^), oxide formation rate: U/UO <0.1 , Th/U = 0.91-0.95 |
| **Data Processing** |  |
| Gas blank | 15 seconds on-peak |
| Calibration strategy | BB1 zircon standard used as primary reference material, Plešovice, KA1 (Kaap Valley tonalite) and SING as secondary reference material (quality control) |
| Reference Material info | BB (Santos et al., 2017): concordia age = 562 ± 0.6 Ma (LA-SF-ICP-MS)  Plešovice (Sláma et al., 2008): 206/238 = 337.13 ± 0.37 Ma (TIMS)  KA1 (Schoene et al., 2006): 207/206 = 3227.2 ± 0.2 Ma (CA-ID-TIMS).  SING (Dunai et al., 2022): 206/238 = 44.80 ± 0.48 Ma (LA-SF-ICP-MS) |
| Data processing package used | in-house EXCEL spreadsheet (Gerdes and Zeh, 2006, 2009) |
| Quality control / Validation | BB (session 1-3): concordia age = 562.32 ± 0.97 Ma (n = 60), MSWD(C+E)=0.57, Prob.(C+E)=1.0  Plešovice (session1-3): concordia age = 337.54 ± 0.61 Ma (n = 57)  MSWD(C+E)=1.03, Prob.(C+E)=0.40  KA1(session 1-3): 207/206 u.i. age = 3227.6 ± 7.3 Ma (n = 42 of 43)  MSWD=0.90, Prob.=0.66  SING (session 1-3): 206/238 w.a.= 44.54 ± 0.14 Ma (n=37)  MSWD=1.07, Prob.=0.35, w.a.= weighted average |

**References**

Dunai, T. J., Binnie, S. A., Gerdes, A, 2022. In situ-produced cosmogenic krypton in zircon and its potential for Earth surface applications, Geochronology, 4, 65–85. https://doi.org/10.5194/gchron-4-65-2022, 2022.

Gerdes, A., Zeh, A., 2006. Combined U–Pb and Hf isotope LA-(MC-)ICP-MS analyses of detrital zircons: Comparison with SHRIMP and new constraints for the provenance and age of an Armorican metasediment in Central Germany. Earth Planet. Sci. Lett. 249, 47–61.

Gerdes, A., Zeh, A., 2009. Zircon formation versus zircon alteration – new insights from combined U–Pb and Lu–Hf in-situ LA-ICP-MS analyses, and consequences for the interpretation of Archean zircon from the Central Zone of the Limpopo Belt. Chem. Geol. 261, 230–243.

Santos, M.M., Lana, C., Scholz, R., Buick, I., Schmitz, M.D., Kamo, S.L., Gerdes, A., Corfu, F., Tapster, S., Lancaster, P., Storey, C.D., Basei, M.A.S., Tohver, E., Alkmim, A., Nalini, H., Krambrock, K., Fantini, C., Wiedenbeck, M., 2017. A new appraisal of Sri Lankan BB zircon as a reference material for LA–ICP–MS U–Pb geochronology and Lu–Hf isotope tracing. Geostand. Geoanalyt. Res. 41, 335–358.

Sláma, J., Kŏsler, J., Condon, D.J., Crowley, J.L., Gerdes, A., Hanchar, J.M., Horstwood, M.S.A., Morris, G.A., Nasdala, L., Norberg, N., Schaltegger, U., Schoene, B., Tubrett, M.N., Whitehouse, M.J., 2008. Plešovice zircon — A new natural reference material for U-Pb and Hf isotopic microanalysis. Chem. Geol. 249 (1-2), 1–35.

Schoene, B., Crowley, J. L., Condon, D. J., Schmitz, M. D., Bowring, S. A., 2006. Reassessing the uranium decy constants for geochronology using ID-TIMS U-Pb data. Geochimica et Cosmochimica Acta 70, 426-445.
